# How Online Food Delivery Services Are Reshaping the Urban Food Environment: Evidence from Hangzhou, China

**DOI:** 10.3390/foods15142481

**Published:** 2026-07-13

**Authors:** Li Chen, Haoying Han, Wanglin Yan, Yang Yang

**Affiliations:** 1 The Institute of Urban and Rural Planning Theories and Technologies, Zhejiang University, Hangzhou 310058, China; 12112027@zju.edu.cn (L.C.);; 2 Institute for Urban and Sustainable Development, City University of Macau, Avenida Padre Tomás Pereira, Taipa, Macau, China; 3 Faculty of Environment and Information Studies, Keio University, 5322 Endo, Fujisawa 252-0882, Kanagawa, Japan

**Keywords:** food environment, OFDS, online–offline, central place theory, Hangzhou

## Abstract

The rapid expansion of Online Food Delivery Services (OFDS) is associated with changes in urban food environments long structured by the central place hierarchy. Does OFDS narrow the spatial inequalities embedded in traditional food access, or reproduce them in a new form? To address the limited multi-dimensional evidence on this question, this study develops an integrated online–offline framework comparing the walkable food environment (W-FE) and the OFDS food environment (OFDS-FE) across three dimensions: accessibility, availability, and affordability. Taking Hangzhou, China as a case, we match multi-source restaurant data via the Hungarian algorithm, model OFDS service ranges with a three-stage Gaussian distance-decay function, and assess spatial equity using the Gini coefficient. Results show that the OFDS-FE is associated with extended potential accessibility beyond walkable catchments and with higher relative affordability of mid- and high-end dining options. At the citywide scale, inequality indicators are lower in the OFDS-FE than in the W-FE, yet disparities persist and, in some suburban areas, widen. All findings are based on modeled, supply-side potential access derived from a single-platform (Meituan), single-month (December 2024) cross-section, rather than on observed consumer behavior. Rather than dissolving the central place hierarchy, OFDS extends the reach of established food centers, forming a broader “digital central service zone.” These findings inform the integration of digital delivery ranges into community life-circle planning.

## 1. Introduction

The urban food environment refers to the broader external conditions through which urban residents access, choose, prepare, and consume food in everyday life. It encompasses physical, economic, sociocultural, and policy dimensions [1,2], and has important implications for dietary health, quality of life, and social well-being [3,4]. In public health and urban geography, food environment equity has been widely recognized as a key mechanism shaping health inequalities [5]. Previous studies have shown that disparities in food environments may influence dietary patterns and energy intake, thereby contributing to chronic disease risks and health disparities [6,7].

In this study, the urban food environment is operationalized as the restaurant-based food-service environment: the set of restaurants and prepared-food services accessible to residents, compared here across its walkable form and its delivery-based form, based on records that can be matched across Dianping and Meituan. It does not cover fresh-food retail, wet markets, supermarkets, household food procurement or home cooking. We retain the broader term “urban food environment” only when referring to the general construct or to the wider literature; all empirical statements in this paper concern the restaurant-based food-service environment. Throughout, all indicators measure supply-side potential access—the restaurant opportunities available to residents under modeled walking or delivery conditions—rather than realized consumer utilization, which additionally depends on individual preferences, time constraints and platform interactions.

**Figure 1:**
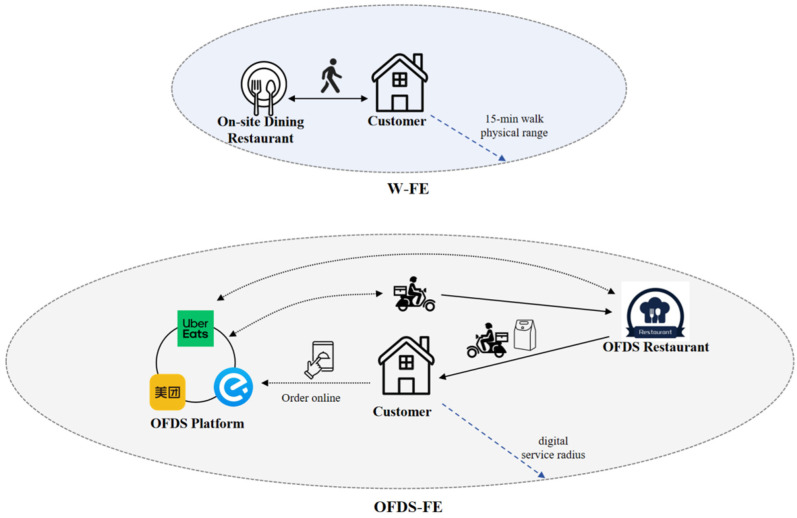
Schematic comparison of the walkable food environment (W-FE) and the online food delivery services food environment (OFDS-FE). The diagram is a conceptual illustration, not an empirical measurement; W-FE indicates restaurant access within residents’ walking catchments, whereas OFDS-FE indicates platform-mediated restaurant access through online ordering, rider dispatch and delivery ranges. Meituan (美团) is shown as an example of a Chinese online food delivery platform.

Within traditional urban food environment research, the walkable food environment (W-FE) has been a key spatial concept. It refers to the food outlets that residents can access within a 10- to 15-min walking distance, including restaurants, supermarkets, and other food-related facilities [8]. The spatial pattern of the W-FE is shaped by geographic location, transport costs, and market rents [9], and often exhibits a central-place structure [10]. Higher-order and more diverse food services tend to concentrate in urban cores, while peripheral areas often have fewer food service options. Such spatial disparities may contribute to the formation of “food deserts” (low healthy food accessibility) or “food swamps” (high unhealthy food exposure) [11,12]. Figure 1 illustrates schematically the contrast examined in this study: the W-FE is bounded by residents’ walking catchments, whereas the OFDS food environment introduced below is bounded by platform delivery ranges centred on the same restaurant supply points.

In recent years, OFDS supported by information and communication technologies have expanded rapidly worldwide. At least one dominant OFDS platform has emerged in countries such as the United States, China and India [13,14,15]. In China, by June 2025, the number of OFDS users had exceeded 592 million, with more than 20 million orders placed per day [15]. The food environment mediated by OFDS, hereafter referred to as the OFDS food environment (OFDS-FE), consists of digital platforms, participating restaurants, and delivery workers. Compared with the W-FE, the OFDS-FE substantially extends the geographic range of food access, with a service radius that can reach up to 10 km [16]. As a result, it may reduce residents’ reliance on food outlets within walking distance and reshape the ways in which food is accessed in cities. Emerging empirical evidence supports this reshaping: online delivery has been found to alter the equity of food accessibility in Nanjing [17], to concentrate online outlet access in the most deprived neighbourhoods of England [18], to extend the geographic reach of food outlets in Canada [16], and to foster “cyber food swamps” in the United States [19].

The expansion of the OFDS-FE may alter the spatial constraints embedded in the traditional service hierarchy [20]. On the one hand, digital platforms and on-demand delivery systems reduce the boundary effects of conventional service areas, enabling residents in low-density or peripheral neighborhoods to access dining options that were previously concentrated in urban cores [17,21]. On the other hand, algorithmic ranking and recommendation, merchant visibility rules, delivery pricing (including minimum-order thresholds) and rider dispatching may generate new spatial biases and potentially reinforce existing inequalities [17] (see Section 2.2). Therefore, OFDS may not simply eliminate spatial disparities in food access, but may instead reorganize them through platform-mediated service areas.

Two research gaps motivate this study. First, online and offline food service systems have rarely been integrated within a single analytical framework; as a result, the structural consequences of OFDS for the overall urban food environment—particularly economic dimensions such as affordability—remain insufficiently understood. Second, the actual service range of OFDS has often been oversimplified as a fixed maximum delivery radius, without considering how service intensity decays over distance. These gaps are elaborated in Section 2.3.

Against this background, this study asks whether and how OFDS is associated with narrower or wider food environment disparities arising from the central-place structure. Specifically, it examines the spatially differentiated effects of OFDS across community types and discusses their implications for spatial equity and community life-circle planning.

Building on central place theory and the platform mechanisms discussed above, the research question is operationalized into four testable hypotheses. H1 (accessibility): the OFDS-FE extends potential food accessibility beyond walkable catchments for all community types, but the magnitude of the extension remains structured by the central place hierarchy, with core areas retaining advantages in higher-order (mid- and high-end) dining categories. H2 (availability): because platform delivery expands demand-side competition faster than restaurant supply, changes in availability are weaker, less consistent and potentially less favorable than the gains in accessibility. H3 (affordability): the OFDS-FE is associated with larger gains in the relative affordability of mid- and high-end dining categories than of affordable everyday categories, for which delivery fees offset low food prices. H4 (equity): at the citywide scale, inequality indicators are lower in the OFDS-FE than in the W-FE (ΔG < 0) in most dimensions, but the equalizing pattern is heterogeneous across dimensions, food categories and community types, and does not dissolve the central–peripheral gradient. These hypotheses are revisited in Section 5 and Section 6.

The remainder of this paper is organized as follows. Section 2 reviews relevant theories and empirical studies. Section 3 introduces the study area, data sources, and data fusion methods. Section 4 presents the research methods and model construction. Section 5 reports the empirical results. Section 6 discusses the findings and planning implications. Section 7 concludes the paper.

## 2. Literature Review

### 2.1. Food Environments

Food environment research has developed a relatively mature theoretical foundation and a substantial body of empirical evidence [21,22]. Existing studies commonly conceptualize the food environment as a multidimensional construct, including interrelated dimensions such as accessibility, availability, affordability, acceptability, accommodation, and sustainability [21]. These dimensions are widely used to assess the ease or difficulty with which residents obtain food. In the physical dimension, accessibility refers to the spatial ease with which residents can reach food outlets near their homes, workplaces, or other activity spaces [23]. Availability refers to the presence and sufficiency of food outlets or food options within a given area, and is often measured by outlet density, counts, or other supply-related indicators [21,22]. Within this framework, the density of food outlets has been widely used as a key indicator, for example, to examine how the distribution of restaurants or fresh food retailers influences food choices [24]. In the economic dimension, affordability refers to the relationship between food prices and residents’ purchasing capacity. It directly affects household food choices, particularly among low-income groups, for whom price can be a major barrier to accessing healthy food [25]. Other dimensions, such as acceptability, accommodation, and sustainability, further reflect residents’ preferences, service convenience, and broader social or environmental concerns [21]. As a result, scholars have increasingly argued that a single-dimensional approach is insufficient to capture the complexity of food environments, and multidimensional measurement frameworks have become common in empirical research.

Building on this multidimensional framework, this study focuses on accessibility, availability and affordability for two reasons. First, these three dimensions can be measured comparably for both the W-FE and the OFDS-FE using restaurant and platform records, whereas acceptability, accommodation, food safety, dietary culture and related dimensions require consumer-, venue- or inspection-level information that is not available at this spatial scale. Second, the three retained dimensions correspond directly to the spatial-economic logic of central place theory: accessibility reflects range, availability reflects threshold demand and supply-demand competition, and affordability reflects price. The inclusion of affordability is especially important because previous OFDS studies have mainly emphasized spatial access, while the economic burden created by restaurant price and delivery fee remains less examined.

A large body of research has shown that food environments exhibit significant socio-spatial disparities and often follow a central-place structure [9]. High-quality and healthy food resources, such as fresh fruits, vegetables, and higher-end restaurants, tend to be concentrated in affluent neighborhoods. By contrast, low-income neighborhoods are more likely to be exposed to dense clusters of low-cost restaurants and energy-dense unhealthy food outlets, resulting in limited access to healthy food and imbalanced nutritional environments [26]. In some urban areas in the United States, residents’ walking distance to food outlets is usually relatively short, typically ranging from 500 m to 1 km, whereas the average distance in suburban areas may exceed 2 km [27]. Such disparities are not limited to Western countries, but are also evident in Asian cities. For example, in Shanghai, access to fresh food is significantly higher in central areas than in non-central areas [28]. In Nanjing, food availability in the main urban area is also higher than that in suburban districts [17]. Similarly, in Tokyo, central areas have a higher density, greater diversity, and better overall quality of restaurants, while peripheral areas are characterized by more limited choices and lower sustainability; these disparities further shape residents’ dining behavior [29,30]. In Hangzhou, residents living in “embedded communities” near affluent neighborhoods may face another form of food access constraint due to the lack of economically affordable dining options [31].

In central place theory terms, each food service is characterized by a range—the maximum distance consumers are willing to travel for it—and a threshold demand—the minimum market size required to sustain it. Services with large thresholds and long ranges (higher-order services, exemplified here by mid- and high-end full-service dining) concentrate in fewer, more central locations, whereas lower-order services with small thresholds (everyday fast-food and deli categories) disperse widely. This spatial–economic logic makes central place theory a natural benchmark for evaluating OFDS: platform delivery changes the effective range of existing restaurants without relocating their supply, so whether the inherited service hierarchy persists under extended ranges becomes a directly testable question. Central place theory does not, however, describe the internal mechanisms of platforms; it is therefore complemented in this study by the platform-mechanism perspective discussed in Section 2.2.

### 2.2. OFDS Food Environment

In recent years, growing scholarly attention has been paid to the impact of OFDS on food environments. Related concepts such as the digital food environment [32] and the OFDS food environment (OFDS-FE) [33] have been proposed. OFDS substantially extends the geographic range of food access and enables consumers to overcome traditional barriers such as adverse weather, limited transportation, and time constraints [34]. It may also reduce exposure to certain health risks in specific contexts [35]. Although food ordering takes place online, the OFDS-FE remains closely connected to the physical environment, as meals are ultimately prepared by brick-and-mortar restaurants and delivered through offline logistics networks [18].

Existing studies on OFDS and food environments can be broadly divided into two methodological streams. The first relies on fieldwork-based approaches, using surveys or interviews to examine OFDS use, food preferences, consumption behavior, and restaurant adaptation [36,37,38,39]. These studies provide detailed insights into food preferences and consumption behavior, but they are often limited to specific samples or study areas and are less able to capture macro-scale spatial patterns. The second stream uses big data and GIS-based methods. These studies collect restaurant information and delivery-related data from OFDS platforms and combine them with geographic information systems and spatial analysis models to measure community-level food availability [17,40,41], restaurant density, and spatial distribution patterns. Such approaches allow for broader spatial coverage, but OFDS data are often difficult to obtain because of privacy and platform restrictions, which limits the completeness and comparability of existing datasets.

Overall, existing studies have mainly focused on the physical accessibility of OFDS, including the proximity and density of online restaurants and the spatial equity of food availability across different areas [17,18,40]. Other studies have examined the potential health implications of OFDS, including nutritional quality and exposure to unhealthy food options [16,19,32]. These studies suggest that OFDS can partly compensate for the limitations of the W-FE, reduce the constraints imposed by location, and expand geographic access in both urban and suburban areas [19,40]. For example, residents with limited access to healthy food may rely more heavily on online food delivery services [40], while the most deprived neighborhoods in the United Kingdom have been found to have the highest level of online food accessibility [18]. However, other studies have also shown that OFDS may widen urban–rural differences in food supply and expand exposure to unhealthy food options, thereby contributing to the growth of “food swamps” [19].

Beyond extending spatial reach, OFDS platforms also mediate food access through internal mechanisms that operate largely out of public view: ranking and recommendation algorithms shape which restaurants consumers encounter first; merchant visibility depends on commission and promotion schemes; delivery pricing changes the effective cost of the same meal across locations and times; and dispatching systems allocate rider capacity over space [17]. Because these parameters are proprietary and unobservable to researchers, spatial studies—including this one—can capture their aggregate spatial outcomes but not their internal logic. A related strand of consumer research further indicates that realized OFDS use depends on individual preferences, time constraints, digital literacy and platform interaction [40], implying a distinction between potential access measured from the supply side and realized utilization observed from behavior [42,43]; the indicators developed in this study belong to the former category. This study therefore treats platform mediation as an explicit interpretive boundary: the measured OFDS-FE reflects the joint outcome of restaurant supply, delivery logistics and platform rules, and algorithmic mediation is flagged as a key caveat in the Discussion and Limitations. What remains unresolved in this literature is how the spatial and the algorithmic faces of OFDS jointly reshape multi-dimensional food access—the integration attempted by the framework developed below.

To make the relationship between the two perspectives explicit: central place theory and the platform-mechanism perspective operate at different levels of the same explanatory structure. Central place theory provides the structural baseline: it explains where restaurant supply is located and why a hierarchical central–peripheral pattern arises, through the two constitutive variables of threshold demand and service range. The platform-mechanism perspective provides the process layer: it explains how the effective range of each existing supply point is produced and differentiated under OFDS—through online ordering, dispatching, delivery pricing and merchant-visibility rules—without relocating supply or changing threshold demand. Platform mechanisms therefore extend central place theory by endogenizing one of its two constitutive variables: range is no longer a fixed distance determined by consumers’ willingness to travel, but a platform-produced, delivery-based decay profile, operationalized as the three-stage distance-decay function in Section 4.2.2. Threshold demand and the location of supply remain governed by the classical central-place logic. This division of labor yields the study’s central testable expectation: if OFDS changes only the range variable, the inherited service hierarchy should persist in higher-order categories even as potential access expands (H1). The concept of the digital central service zone (Section 6.2) names the resulting structure, in which platform-extended ranges radiate from, rather than dissolve, the existing central places.

### 2.3. Research Gaps and Analytical Framework

Previous studies have shown that the W-FE often follows a central-place structure. Food access in central areas is generally easier, with shorter distances and lower time costs, whereas peripheral areas usually require longer travel distances and higher time costs. Studies on the OFDS-FE have further shown that the expansion of OFDS may increase exposure to unhealthy food options [19]. Other research has examined the relationship between the built environment and dining mode choice, suggesting that residents with fewer walkable food options are more likely to use OFDS [40]. However, existing studies have not systematically answered whether the OFDS-FE reshapes food environment disparities among different areas that are produced by this central–peripheral gradient, nor whether it reduces the multidimensional costs of food access in peripheral communities and thereby improves spatial equity in the urban food environment.

Two major research gaps can be identified. First, existing studies have rarely integrated online and offline food service systems within a unified analytical framework. As a result, the structural effects of OFDS on the overall urban food environment remain insufficiently understood. This also leads to a relatively narrow focus on the physical dimension of OFDS, particularly spatial accessibility, while economic dimensions such as affordability have received less attention. Second, the actual service range of OFDS has often been simplified. Many studies define OFDS service areas according to the maximum delivery radius provided by platforms, without considering how service intensity declines with distance.

**Figure 2:**
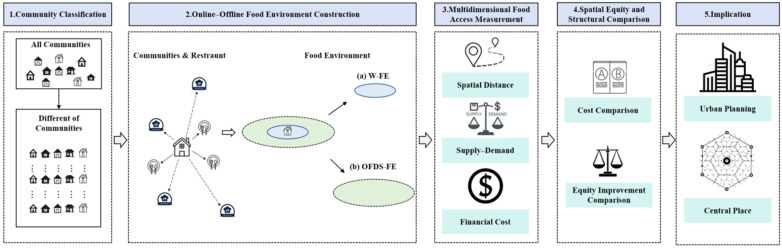
Online–offline integrated analytical framework used in this study. The framework classifies residential communities, constructs the walkable food environment (W-FE) and the online food delivery services food environment (OFDS-FE), measures accessibility, availability and affordability, and evaluates spatial equity and central-place implications. The framework is conceptual and is operationalized with community, Dianping and Meituan data described in Section 3.

To address these limitations, this study develops an integrated online–offline analytical framework (Figure 2). Excluding dimensions such as food safety and dietary culture, the framework compares the ease of accessing the same food categories from the W-FE and the OFDS-FE at the community scale. This design allows us to examine whether OFDS is associated with changes in spatial disparities in food environments that arise from the central-place structure. First, communities in the study area are classified according to their socioeconomic and spatial attributes. Second, restaurant point-of-interest (POI) data are used to construct the W-FE and OFDS-FE for each community, with particular attention to the delineation of service ranges. Third, mathematical models are applied to measure the ease of accessing different food categories across multiple dimensions. Finally, the results are used to discuss the implications of OFDS for community planning in Hangzhou. In sum, the distinctive contribution of this framework is that it measures the walkable and the delivery-based food environments on the same three dimensions, at the same community scale and for the same food categories, and replaces the fixed-radius delivery assumption with a delivery-process-based service-range model. This design makes it possible to test directly whether platform delivery reshapes, rather than merely overlays, the central-place pattern of restaurant access.

## 3. Study Area and Data

### 3.1. Study Area and Conceptual Definitions

**Figure 3:**
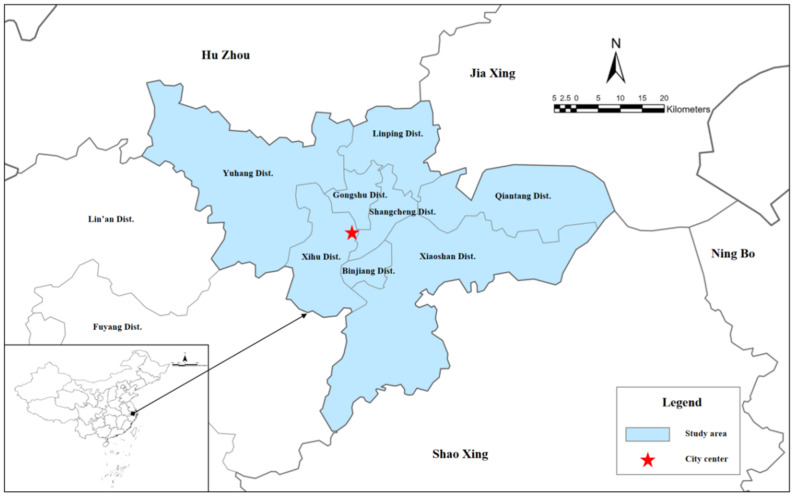
Study area in Hangzhou, China. The blue polygons indicate the eight main urban districts included in the analysis, and the red star marks the city center used to calculate community distance to the center. The map is based on administrative boundary data and is used to define the spatial scope of the community-, restaurant- and platform-based measurements.

This study uses Hangzhou, Zhejiang Province, China, as the case study. Hangzhou has a high penetration rate of OFDS and provides rich platform-based data, making it a suitable case for examining the interaction between OFDS and the urban food environment. The study area focuses on the main urban area of Hangzhou, including eight administrative districts: Xihu, Gongshu, Shangcheng, Binjiang, Xiaoshan, Yuhang, Qiantang, and Linping (Figure 3). In 2022, the permanent population of the study area reached 9.221 million, accounting for 74.5% of Hangzhou’s total population [44]. The study area also contained 82.1% of the city’s OFDS restaurants and accounted for 84.3% of its OFDS orders. It therefore represents the core area of urban functions and population activities in Hangzhou and provides an appropriate spatial setting for analyzing the relationship between OFDS and the urban food environment. The remaining county-level administrative divisions of Hangzhou outside the study area—Fuyang District, Lin’an District, Tonglu County, Chun’an County and Jiande City (a county-level city)—were excluded because they are spatially discontinuous from the contiguous main urban area, are predominantly rural or mountainous, and would blur the central–peripheral gradient examined in this study. Hangzhou is broadly representative of large, high-penetration Chinese platform-economy cities: it is a new first-tier megacity with polycentric expansion and mixed housing vintages, conditions under which central–peripheral contrasts in food services are clearly observable. At the same time, as the headquarters city of Alibaba, its digital-service intensity is above the national average; this particularity is treated as a boundary condition for generalization in the Discussion (Section 6.4).

This study uses residential communities as the basic unit of analysis. A residential community refers to a residential compound with a clearly defined spatial boundary, consisting of housing buildings and associated public spaces. It is the basic spatial unit of residents’ daily life and the most direct spatial carrier through which food services and OFDS affect residents. Restaurants are classified into two types. The first type is offline dine-in restaurants, referring to brick-and-mortar restaurants that provide on-site dining services. The second type is OFDS restaurants, referring to restaurants that provide food delivery services through online platforms. Accordingly, the W-FE refers to the food environment formed by offline dine-in restaurants within the walking range of a residential community, whereas the OFDS-FE refers to the food environment formed by OFDS restaurants that can deliver to that community within the platform-defined service range.

### 3.2. Data and Processing

#### 3.2.1. Data Sources

This study uses multi-source heterogeneous data. The OFDS restaurant data were obtained from Meituan, the online food delivery platform with the largest market share in China. Recent market reporting citing BOCOM International estimated that Meituan accounted for approximately 65% of China’s food-delivery market by transaction share in 2024, compared with approximately 33% for Ele.me and 2% for other platforms [45]. The data were collected in December 2024 and include restaurant name, address, geographic coordinates, cuisine category, monthly sales, average spending per person (per-capita consumption), delivery fee, and average delivery time. After initial collection, the dataset contained 32,289 records. Because the data were collected in December, when colder weather is associated with seasonally elevated delivery demand [34], the measured intensity of OFDS activity may be somewhat higher than the annual average; all indicators in this study are accordingly based on a single cross-section.

The offline dine-in restaurant data were obtained from Dianping POI data for the same period. The dataset includes restaurant name, address, geographic coordinates, cuisine category, and average price per person (per-capita consumption), with a total of 50,025 records. Residential community data were collected from Anjuke, a major real estate platform in China. These data include community name, address, year of construction, number of households, floor area ratio, property management fee, and average housing price in 2024.

#### 3.2.2. Data Processing and Integration

According to the regulations of the Hangzhou Municipal Administration for Market Regulation, restaurants providing OFDS must have a physical offline storefront [46]. Therefore, OFDS restaurants can be regarded as a subset of offline dine-in restaurants. Under this rule, licensed cloud or shared kitchens hold physical premises, appear in the data as ordinary supply points, and are retained; what the matching cannot identify are multi-brand virtual storefronts operating from a single licensed kitchen, which may modestly inflate the count of nominal online restaurants. This boundary is acknowledged in Section 6.4. To distinguish restaurants that only provide offline dine-in services from those that also provide OFDS, avoid duplicate counting, and integrate offline and online consumption attributes from different platforms, it is necessary to match restaurant records from Dianping and Meituan with high precision.

Restaurant and community records were collected in December 2024 through batch collection of publicly displayed listing pages, covering the fields listed in Table 1.

Before matching, both datasets went through a common cleaning procedure. Records were deduplicated on the combination of restaurant name, address and coordinates; obviously non-restaurant and permanently closed records were removed; coordinates were converted to a unified coordinate system and checked against the study-area boundary, with implausible locations re-geocoded from their addresses; and records missing any core matching attribute (name, coordinates or category) were excluded.

**Figure 4:**
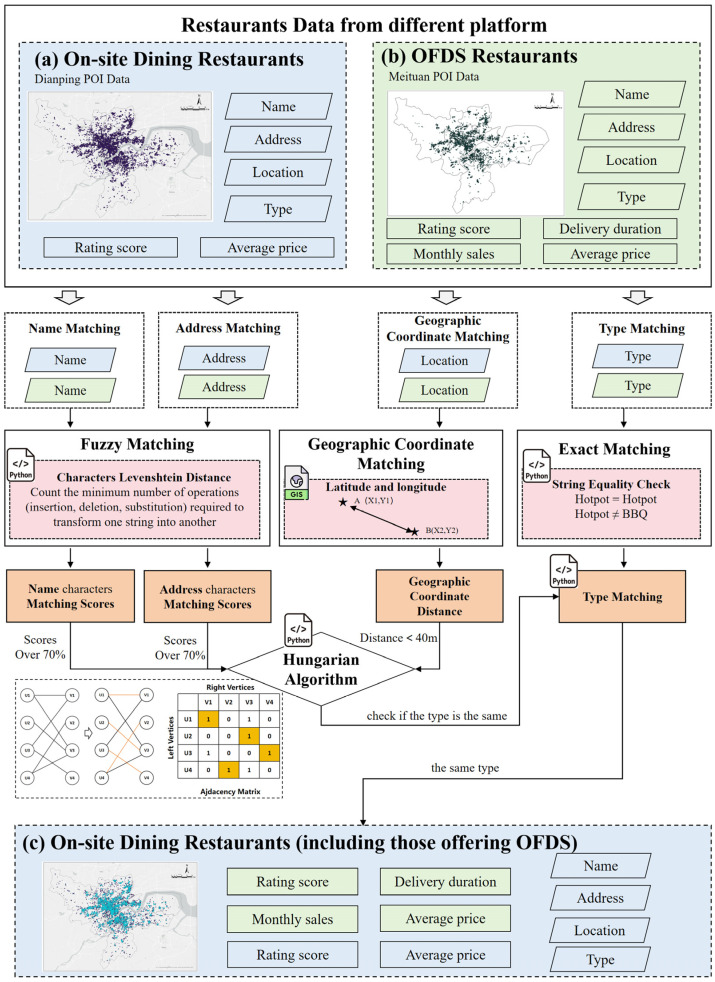
Optimal matching framework for integrating Dianping offline dine-in restaurant records and Meituan online food delivery services (OFDS) restaurant records. Matching uses restaurant name, address, geographic coordinates and category information, with fuzzy text matching, geographic compatibility and type matching combined through the Hungarian algorithm. Input records are observed platform/listing data collected in December 2024; the matched pairs are model-derived integration results.

This study develops an optimal matching framework based on the Hungarian algorithm (Figure 4) to integrate online and offline restaurant data from Meituan and Dianping. The Hungarian algorithm is a classic combinatorial optimization method for solving maximum-weight bipartite matching problems. It enables globally optimal one-to-one matching and maximizes overall similarity across matched records [47].

Both datasets contain four core restaurant attributes: restaurant name, address, geographic coordinates, and cuisine category. These attributes were used as matching criteria. Restaurant names and addresses were processed using fuzzy matching, with text similarity calculated based on the Levenshtein distance. Geographic coordinates were used to calculate spatial distance and ensure locational proximity. Cuisine category was treated as an exact matching criterion, requiring candidate records to belong to the same food category in order to reduce cross-category matching errors. In total, 92% of the Meituan records (29,641 of 32,289) were successfully matched to Dianping records; unmatched records were excluded from the subsequent analysis.

**Figure A1:**
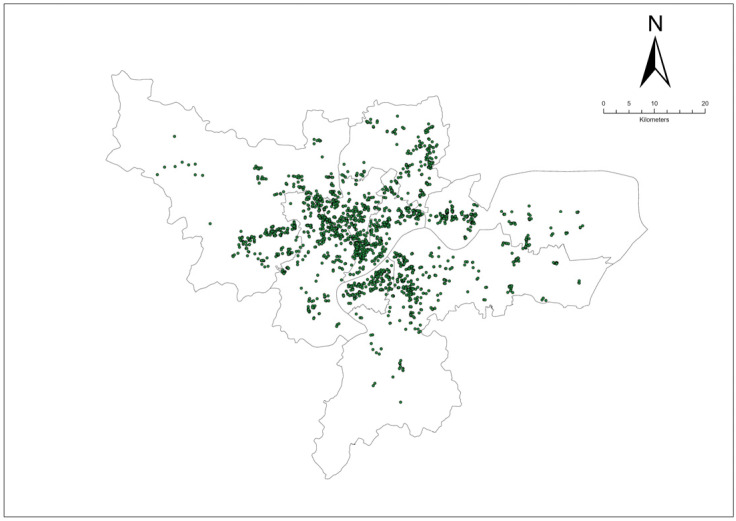
Spatial distribution of unmatched Meituan restaurant records.

To assess whether the cross-platform matching procedure introduced major classification errors, we conducted a manual validation exercise (Appendix B). A stratified random sample of 500 matched Meituan-Dianping pairs was independently checked using restaurant name, brand, address, category and coordinate proximity. Among these pairs, 498 were confirmed as correct matches and two remained ambiguous after double checking, yielding a conservative precision estimate of 99.6% if ambiguous cases are treated as non-confirmed matches. We also checked 200 sampled unmatched Meituan records; none was judged to have a corresponding Dianping record in the sampled validation. In addition, the spatial distribution of unmatched Meituan records was mapped in Appendix B (Figure A1); these records were dispersed across the main urban area and nearby suburban commercial nodes, rather than concentrated in a single peripheral zone. These checks suggest that the exclusion of unmatched records is unlikely to introduce a strong directional spatial bias, although residual undercoverage is acknowledged as a limitation.

#### 3.2.3. Restaurant Classification

Restaurant types differ substantially in menu composition, dining duration, service format, and price level, and these differences directly affect residents’ food choices and willingness to pay [48]. Therefore, after cross-platform matching, restaurant categories from Meituan and Dianping were further standardized to ensure the comparability between the W-FE and the OFDS-FE. If the original platform labels were used directly, differences in category granularity between the two platforms could lead to biased estimates of accessibility, availability, and affordability.

Based on cuisine characteristics, dining duration, service format, and average price, all restaurants were first classified into two broad groups: full-service restaurants and fast-food and snack restaurants. They were then further divided into 25 subcategories (Table 2). Full-service restaurants generally have higher prices, longer dining durations, and stronger experiential or social attributes, whereas fast-food and snack restaurants are usually more standardized, lower-priced, and more dependent on physical proximity. This classification provides the basis for setting differentiated service ranges and distance-decay parameters in the following measurement.

For the core comparative analysis, this study selected six representative restaurant categories: Jiangsu–Zhejiang cuisine, Sichuan–Hunan cuisine, Japanese cuisine, rice noodles and wheat noodles, rice set meals, and braised and marinated deli foods. The selection was based on five criteria: sufficient observations in both offline and OFDS datasets, broad spatial coverage across Hangzhou, clear daily meal function, comparability between offline and online service forms, and relatively stable consumption demand. These six categories also represent different price levels and dining scenarios, ranging from medium- and high-end full-service restaurants to low-cost everyday meals. Their customer demand and service modes in offline dine-in and OFDS contexts were further compared [49].

Among the six selected categories, Jiangsu–Zhejiang cuisine and Sichuan–Hunan cuisine represent common medium- to high-end full-service restaurants in Hangzhou. In offline dine-in settings, these categories usually involve table service, regional flavors, and a relatively strong dining-experience component [50]. In the OFDS context, they are often adapted into cooked dishes or set meals suitable for delivery [51]. Japanese cuisine is included as a high-end full-service category [52]. Its offline consumption is more strongly associated with food freshness, dining environment, and service quality, whereas OFDS offerings are mainly delivered in the form of sushi, sashimi, and bento-style meals. These three categories therefore represent full-service dining with different levels of price and experiential dependence.

Rice noodles and wheat noodles, rice set meals, and braised and marinated deli foods represent fast-food and snack categories. Rice noodles and rice set meals are relatively affordable, standardized, and frequently consumed in daily meals, with shorter dining durations and smaller walking service ranges [51]. In the OFDS context, their online and offline food forms are highly comparable because they are commonly provided as standardized staple meals. Braised and marinated deli foods are also included because they usually do not rely on table service and have ready-to-eat characteristics. Therefore, the offline purchase and OFDS forms of this category are largely consistent.

**Figure 5:**
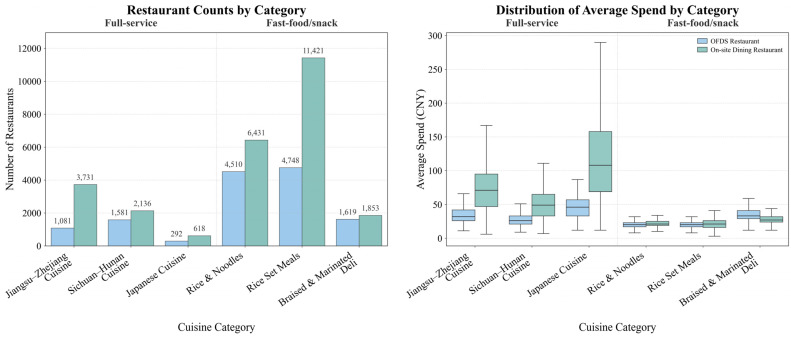
Comparison of restaurant counts and average prices for the six analytical categories. Bars show observed restaurant counts from matched Dianping offline records and Meituan OFDS records, and boxplots show observed average spending per person in CNY/person. Categories include three full-service restaurant groups and three fast-food or snack groups used in the core online–offline comparison.

Other restaurant categories were retained in the classification system but excluded from the core comparison for three main reasons. First, some categories had insufficient OFDS observations, such as private dining, beef and mutton hotpot, and grilled meat. Second, some categories were internally heterogeneous, such as other regional cuisines and specialty snacks, making it difficult to define a consistent service logic. Third, some categories were strongly affected by dine-in experience, seasonality, group dining, or time-specific consumption, such as hotpot, barbecue, crayfish and grilled fish, seafood, and breakfast foods. Including these categories in the core comparison could reduce the comparability between the W-FE and the OFDS-FE and introduce bias into the measurement of spatial access and affordability. As shown in Figure 5, the six retained categories account for 52.4% of offline restaurant records (26,190 of 50,025), 46.7% of matched online restaurants (13,831 of 29,641), and 47.9% of the recorded monthly sales volume on Meituan under lower-bound counting of banded entries (e.g., “monthly sales 200+” counted as 200 orders; 12,052,310 of 25,151,796). The core comparison therefore covers roughly half of the restaurant food environment and spans both everyday staple segments and full-service dining, while the excluded categories are mostly scenario-based, time-specific or internally heterogeneous, as summarized in Table 2. The six categories therefore provide a suitable basis for comparing how the W-FE and the OFDS-FE differ in accessibility, availability, and affordability across community types.

## 4. Research Methods

### 4.1. Measurement Indicators

This study uses three core indicators—accessibility, availability, and affordability—to measure differences in community food environments (Table 3).

Accessibility measures the spatial convenience with which residents can access food service resources. It is represented by the distance-weighted number of restaurants available to a community. A higher value indicates lower spatial impedance and a wider range of restaurant choices.

Availability captures the supply–demand relationship within the food environment, reflecting the relative match between community population size and restaurant supply. It is measured by the distance-weighted number of restaurants per capita. A lower value indicates tighter restaurant supply relative to population demand.

Affordability measures residents’ relative purchasing capacity for dining services. It is represented by the ratio of residents’ daily living expenditure to the distance-weighted average restaurant price. A higher value indicates a lower economic burden and stronger affordability for accessing a given type of food service. Daily living expenditure E is derived from the per-capita annual consumption expenditure of residents reported in the Hangzhou Statistical Yearbook (50,129 CNY per year, approximately 137 CNY per day) [44] and is applied as a citywide constant; cross-community differences in affordability therefore reflect differences in distance-weighted dining costs rather than assumed income differences.

### 4.2. Food Environment Scope and Analytical Models

#### 4.2.1. Spatial Scope of the Food Environment

The spatial scope of the W-FE is defined based on a 15-min walking distance from each residential community, corresponding to a 1.5 km straight-line buffer. Restaurants located within this range are included in the W-FE. Considering that residents generally have a shorter willingness-to-walk distance for fast-food and snack restaurants than for full-service restaurants with better dining environments and more distinctive cuisines, this study further differentiates the walking catchment by restaurant type. Specifically, full-service restaurants within 1.5 km and fast-food and snack restaurants within 800 m of each residential community are included in the W-FE measurement. These thresholds follow purpose-stratified walking-distance evidence and Chinese community life-circle planning standards, which treat full-service dining as a 15-min destination and everyday fast food as a short-range daily need [8,53].

The spatial scope of the OFDS-FE depends on the service radius defined by online food delivery platforms. In this study, 8 km is used as the outer Meituan delivery range observed in the data-collection setting. This value defines the maximum platform service boundary for constructing the OFDS-FE, while the delivery-based distance-decay function in Section 4.2.2 is used to represent how effective service intensity declines within that boundary.

#### 4.2.2. Distance Decay Effects

**Figure 6:**
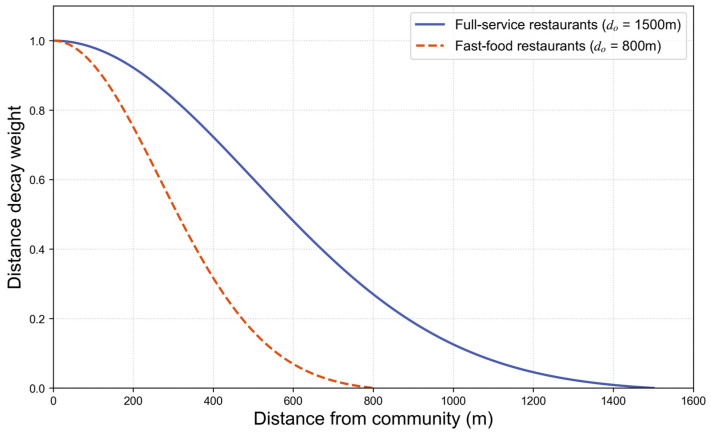
Gaussian distance-decay functions for offline dine-in restaurants in the walkable food environment (W-FE). The horizontal axis reports distance from the community in meters, and the vertical axis reports the modeled distance-decay weight (DW). Full-service restaurants use d0 = 1500 m, while fast-food and snack restaurants use d0 = 800 m.

As distance increases, the service influence of restaurants gradually declines. Previous studies commonly use a Gaussian monotonic decay function to represent the weakening of facility service intensity with distance, because this function can continuously capture how the influence of commercial facilities decreases over space [52]. Accordingly, this study uses a Gaussian decay function to model the decline in the service influence of offline dine-in restaurants with increasing distance from residential communities. The distance threshold is set at 1.5 km for full-service restaurants and 800 m for fast-food and snack restaurants (Figure 6, Equation (1)).

(1) $$G_{W-FE}(d_{ic})\left\{\begin{array}{c} DW=\frac{e^{-\frac{1}{2}\times (\frac{d_{ic}}{d_{o}}{)}^{2}}-e^{-\frac{1}{2}}}{1-e^{-\frac{1}{2}}\;}\;\;\;\;\;\;{(d}_{ic}\leq d_{o})\\ DW=0\;\;{\;\;(d}_{ic}>d_{o}) \end{array}\right.$$

(2) $$G_{OFDS-FE}(d_{ic})\left\{\begin{array}{c} \;\;\;\;\;DW=1,\;({\;d}_{ic}<2\;km)\\ \;\;\;DW=\frac{e^{-\frac{1}{2}\times (\frac{d_{ic}}{d_{o}}{)}^{2}}-e^{-\frac{1}{2}}}{1-e^{-\frac{1}{2}}\;}\;\;\;\;\;\;{(8\;km>d}_{ic}\geq \;2\;km)\\ DW=0,\;({\;d}_{ic}\geq 8\;km) \end{array}\right.$$

However, the service process of OFDS differs fundamentally from offline dine-in services: consumers place orders online, while delivery workers complete offline pickup and delivery. Therefore, a single monotonic Gaussian decay function is insufficient to capture the full service process of OFDS. To more accurately represent how OFDS service intensity changes with distance, this study constructs a delivery-based distance decay function by considering the process of “online ordering–platform dispatching–restaurant pickup–meal delivery.”

**Figure 7:**
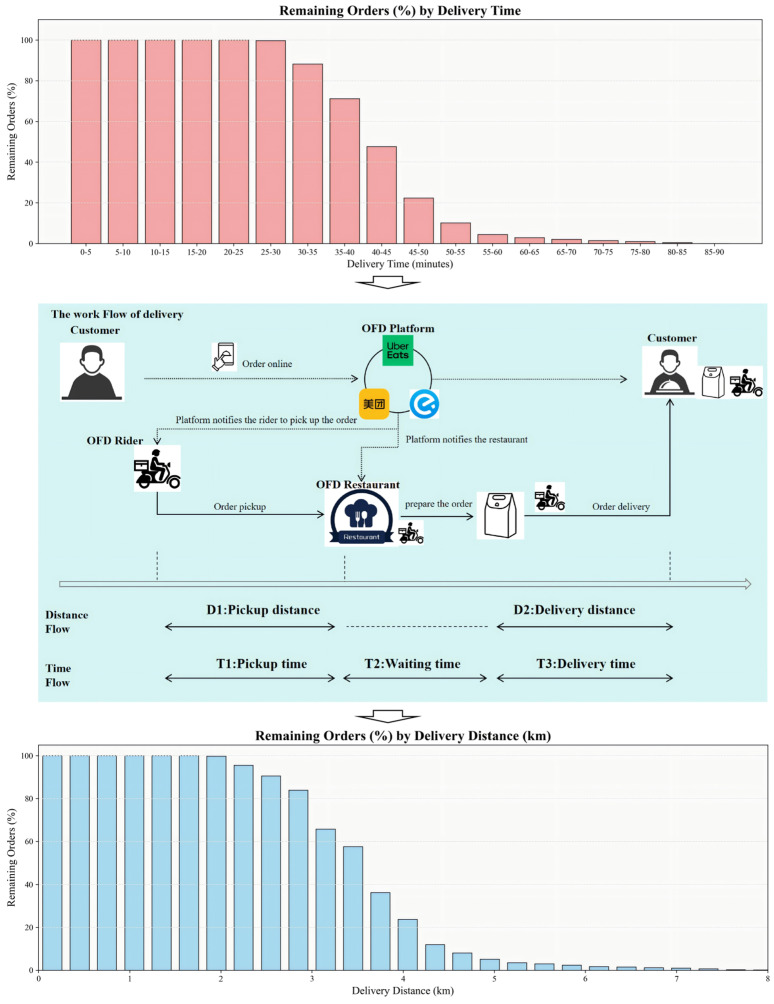
OFDS delivery workflow and the transformation from delivery time to estimated delivery distance. OFDS denotes online food delivery services. The upper chart summarizes observed Meituan (美团) delivery-time records, the middle panel shows the online ordering, dispatching, restaurant pickup and delivery process, and the lower chart shows modeled delivery-distance distribution derived from delivery time using the assumptions in Section 4.2.2. Time is measured in minutes and distance in kilometers.

Because OFDS platforms do not provide the actual delivery distance between restaurants and consumers, this study estimates delivery distance from platform-recorded delivery time (Figure 7). Based on the standard delivery service process described [54] and Meituan’s algorithm disclosure on rider waiting time [55], the total delivery time is decomposed into pickup travel time T1, waiting time T2, and delivery travel time T3. Since detailed rider trajectories are unavailable, pickup and delivery travel times are assumed to have similar overall distributions, namely T1 = T3. The restaurant waiting time is approximated as 10 min, namely T2 = 10 min, according to the rider waiting-time information reported by Meituan [55]. The delivery distance is therefore estimated as:

$$d_{ic}=(T_{total}-T_{2})v/2$$

where dic denotes the estimated delivery distance between community i and restaurant c, Ttotal is the platform-recorded total delivery time, T2 is the waiting time, and v is the average cycling speed of delivery workers, set at 15 km/h [54]. The delivery-time data are thus converted into estimated delivery distances, which are then used to fit the OFDS distance decay function.

**Figure 8:**
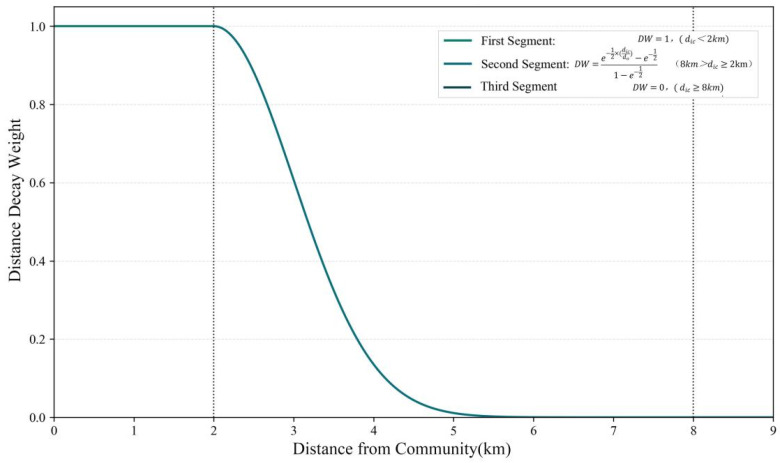
Three-stage distance-decay function for the online food delivery services food environment (OFDS-FE). The horizontal axis reports delivery distance from the community in kilometers, and the vertical axis reports the modeled distance-decay weight (DW). The function assigns full weight within 2 km, applies Gaussian decay between 2 km and 8 km, and assigns zero weight beyond the 8 km Meituan service boundary observed in the data-collection setting.

The fitted function shows a three-stage pattern (Figure 8, Equation (2)). When dic < 2 km, the distance decay weight is set to 1, indicating that short-distance delivery is not substantially constrained by distance. When 8 km > dic ≥ 2 km, the weight follows a Gaussian monotonic decay pattern, reflecting the decline in service accessibility as delivery distance increases. When dic ≥ 8 km, the weight is set to 0, indicating that the restaurant is beyond the regular OFDS service range and cannot provide delivery service to the community.

The OFDS service boundary and the effective distance-decay parameter play different roles. The 8 km value is used as the outer Meituan delivery range observed in the data-collection setting, whereas the distance-decay function estimates how effective service intensity declines within that outer boundary. Using the observed order-duration sample (*n* = 36,793), $T_{2}$ = 10 min and *v* = 15 km/h, the fitted decay parameter is *d*0 = 1.42 km (R^2^ = 0.983), corresponding to a half-decay distance of approximately 3.2 km. This parameter implies that nearby restaurants dominate the effective OFDS choice set even when the platform’s formal service radius extends farther. The shares of implied delivery distances within 5 km and 8 km are reported as sensitivity diagnostics in Appendix C, and comparisons with alternative decay-function families are reported in Appendix D. In terms of the theoretical framework (Section 2.2), this delivery-based decay function operationalizes the platform-produced range through which OFDS extends the central-place logic; it is the empirical bridge between central place theory and the platform-mechanism perspective.

#### 4.2.3. Calculation Methods

This study uses a distance-decay-weighted gravity accessibility model to calculate accessibility and affordability, and applies the enhanced two-step floating catchment area method (E2SFCA) to calculate availability (Appendix A.1). Both methods incorporate the distance decay function $G(d_{ic})$. The E2SFCA method further accounts for both facility supply and population demand, integrating spatial distance and service capacity, and is therefore suitable for measuring service availability [56,57]. In implementing the E2SFCA, the supply capacity of each restaurant is assumed to be sufficient relative to demand and is therefore set as identical across restaurants (Appendix A.1); availability thus reflects the spatial configuration of supply relative to population demand rather than differences in individual restaurant capacity.

We retained this symmetric equal-capacity specification because comparable capacity indicators are not available for offline and online restaurants. Monthly sales are observed only for Meituan stores and measure realized demand rather than supply capacity; using them as capacity weights would therefore make the online numerator partly endogenous to the outcome being evaluated and would break comparability with the offline food environment. Sales-weighted availability is consequently not reported as a robustness test, and future work should revisit capacity weighting when comparable seating, floor-area, staffing or order-capacity information is available for both offline and online restaurants.

In the W-FE, the three indicators are calculated as follows. First, accessibility is measured using the distance-decay-weighted gravity accessibility model. Restaurants within the walking catchment are assigned weights according to $G(d_{ic})$, including full-service restaurants within 1.5 km and fast-food and snack restaurants within 800 m of each community. Second, availability is calculated using the E2SFCA method. In the first step, the population covered by each restaurant’s walking service area is aggregated with the same distance decay weights to obtain a restaurant-level supply–demand ratio. In the second step, these ratios are weighted by distance and aggregated back to each community to obtain community-level availability. Third, affordability is measured by calculating the distance-weighted average price of accessible restaurants and then dividing residents’ daily living expenditure by this weighted average price.

The accessibility indicator should therefore be interpreted as a distance-decay-weighted measure of potential physical access from residential communities to restaurant services. It captures spatial proximity and service-range effects, but it does not directly measure restaurant quality, seating capacity, opening hours, hygiene conditions or consumer preferences. These dimensions are difficult to define consistently from the available platform records and are therefore treated as data and conceptual limitations rather than as components of the accessibility score.

In the OFDS-FE, the calculation logic is generally consistent with that of the W-FE. The main differences lie in the service range and the distance decay function. The OFDS-FE uses the platform-based delivery service range, and both full-service and fast-food restaurants are weighted using the delivery-based 2–8 km segmented distance-decay function. For affordability, the monetary cost of each OFDS restaurant is measured as the average food price plus the delivery fee, rather than the food price alone. This total delivered cost is then used to calculate the distance-weighted average cost and compare it with residents’ daily living expenditure. Based on this approach, accessibility, availability, and affordability are calculated separately for the OFDS-FE (Table 4).

### 4.3. Community Classification

**Figure 9:**
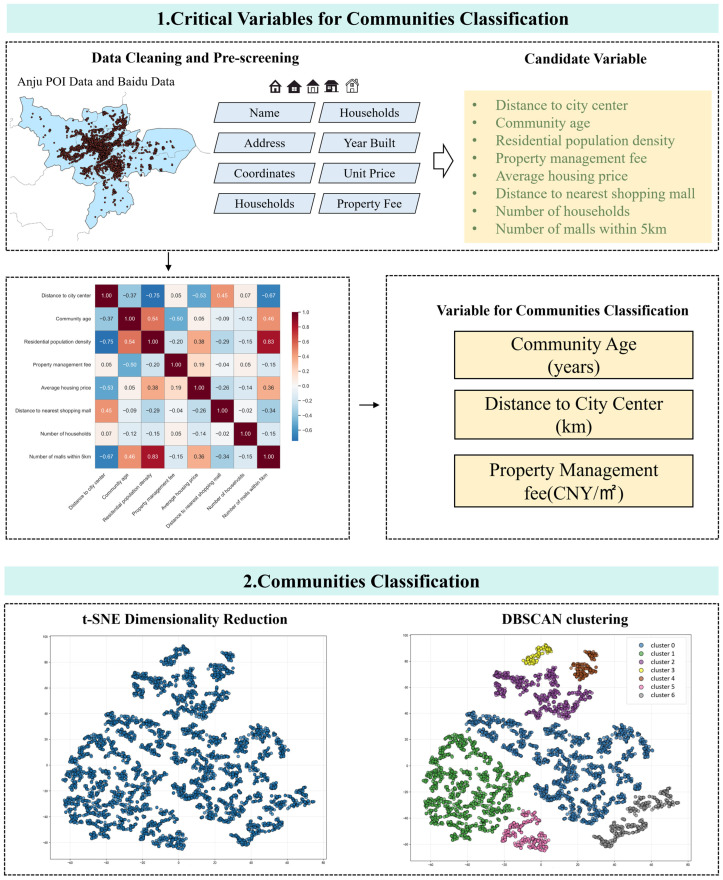
Community classification framework for residential communities in Hangzhou. Candidate variables come from community records and spatial measures; the final classification uses community age (years), distance to city center (km) and property management fee (CNY/m^2^). t-SNE dimension reduction and DBSCAN clustering produce seven descriptive community clusters, whose substantive meanings are reported in Table 5.

Before examining the associations between OFDS and the urban food environment, this study first classifies residential communities in Hangzhou to identify potential differences in their socioeconomic and spatial attributes (Figure 9). The original community dataset includes multiple variables, such as housing price, building age, number of households, and floor area ratio. During data preprocessing, missing values were imputed and outliers were removed. Additional spatial variables were also calculated, including distance to the city center, residential population density, distance to the nearest large shopping mall, and the number of shopping malls within a 5 km radius.

In the variable selection process, correlation analysis and multicollinearity tests were used to identify three core indicators that independently capture community differences: (1) distance to the city center, measured in kilometers, representing locational conditions; (2) building age, measured in years, representing the renewal status of the community; and (3) property management fee per square meter, reflecting housing-market and service-fee stratification. These three indicators have relatively low correlations with one another, which helps reduce redundant information and multicollinearity while jointly describing communities from spatial and economic perspectives.

For community clustering, this study uses a nonlinear dimension-reduction and density-clustering pipeline. Building age, distance to the city center and property management fee were mean-imputed, transformed where necessary and z-standardized. t-SNE was used to project the three-dimensional attribute space into two dimensions (perplexity = 40, PCA initialization and fixed random seed) [58], and DBSCAN was then applied to the embedding (MinPts = 25; epsilon = 4.2, located at the elbow of the k-distance curve) [59]. The resulting seven groups are interpreted as descriptive strata along a continuous age-fee-location gradient rather than as compact natural classes. This resolution was retained because it balanced interpretability and granularity: smaller epsilon values fragmented the embedding into many local groups or noise points, whereas larger epsilon values merged communities with distinct locational, age and property-fee profiles. The seven-cluster solution therefore captures the main community profiles needed for comparison while avoiding excessive fragmentation. Parameter perturbations affect exact boundaries, but the substantive central-peripheral gradients and cluster-level conclusions remain stable, as reported in Appendix G.

Based on the mean and median values of the three core indicators, the seven community clusters are defined as follows: Cluster 0 represents non-central older low-fee communities; Cluster 1 represents non-central newer high-fee communities; Cluster 2 represents central older mid-fee communities; Cluster 3 represents central old low-fee communities; Cluster 4 represents central oldest communities; Cluster 5 represents suburban newer high-fee communities; and Cluster 6 represents suburban older low-fee communities (Table 5).

### 4.4. Equity Measurement

To evaluate the spatial equity of the improvement associated with OFDS, this study uses the Gini coefficient G as a quantitative measure. The Gini coefficient was originally developed to measure inequality in income and wealth distribution [60]. It has since been widely applied to health care [61], education [62], public facilities, and other fields to assess the distributional equity of resources. In this study, the Gini coefficient is used to measure disparities in food environments across communities, including three dimensions: accessibility, availability, and affordability.

To examine whether the OFDS-FE exhibits lower inequality than the W-FE, this study calculates the Gini coefficients of the W-FE and the OFDS-FE separately, denoted as $G^{W-FE}$ and $G^{OFDS-FE}$. Their difference is defined as:

$$∆G=G^{OFDS-FE}-G^{W-FE}$$

If ∆G < 0, inter-community disparities are smaller in the OFDS-FE, indicating higher spatial equity. If ∆G>0, the OFDS-FE increases disparities and weakens spatial equity. If ∆G=0, the OFDS-FE has no measurable effect on spatial equity.

In the analytical framework, this study calculates the Gini coefficient not only for all communities, namely All Communities (Clusters 0–6), but also for six selected comparison groups. These groups are designed to distinguish the relative roles of locational conditions and fee-and-age attributes in shaping the equity of restaurant-service access.

First, three locational comparison groups are constructed under broadly comparable property-fee and building-age conditions: Loc-MidFee (Clusters 0 and 2), Loc-HighFee (Clusters 1 and 5), and Loc-LowFee (Clusters 3 and 6). These groups compare communities with broadly similar property-fee and building-age profiles but different locational conditions, thereby identifying how central, non-central, and suburban locations affect the equity of restaurant-service access.

Second, three fee-and-age comparison groups are constructed under similar locational conditions: Fee-Central (Clusters 2, 3, and 4), Fee-Noncentral (Clusters 0 and 1), and Fee-Suburban (Clusters 5 and 6). These groups compare communities with different property-fee and building-age characteristics within the same broad locational context. These stratified comparisons are not intended as strict causal controls, but rather as a way to identify how the equity effects of the OFDS-FE vary across communities with different spatial and socioeconomic attributes.

## 5. Results

### 5.1. Measurement Results of Food Environments

Unless noted otherwise, all W-FE versus OFDS-FE contrasts described in this section are statistically significant in paired Wilcoxon signed-rank tests with Benjamini–Hochberg correction across the 18 dimension–category combinations (all adjusted *p* < 0.001); test statistics, effect sizes and directions are reported in Appendix E.

**Figure 10:**
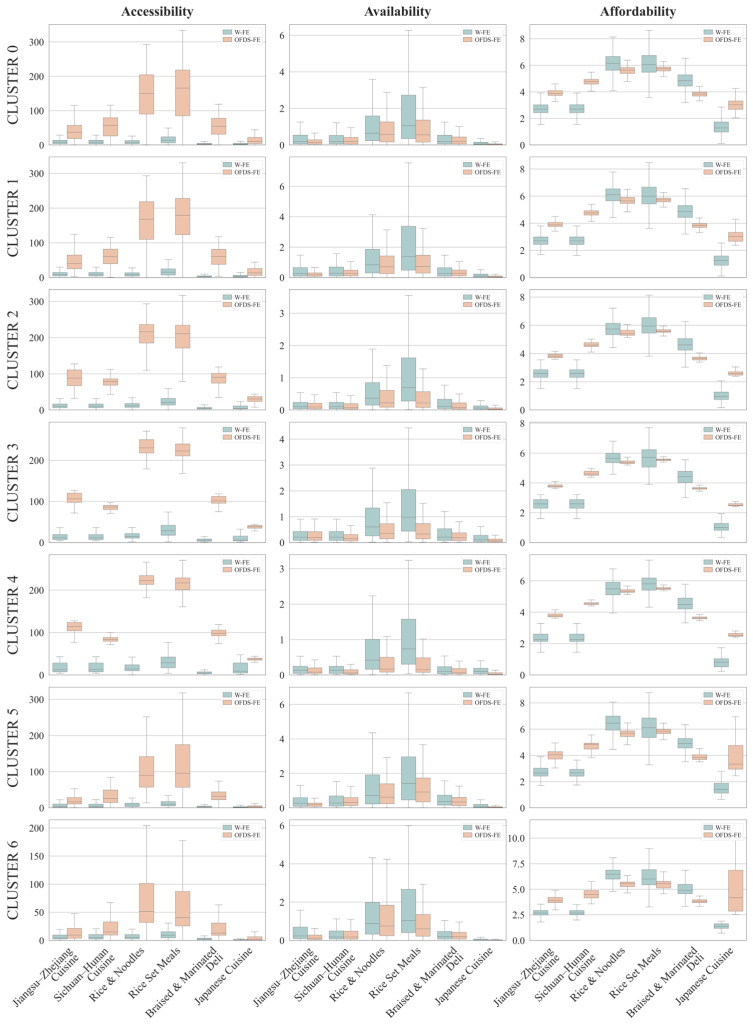
Comparison of the walkable food environment (W-FE) and the OFDS food environment (OFDS-FE) across the seven community clusters defined in Table 5. Boxes show community-level distributions of modeled, supply-side indicators (accessibility, availability, affordability) for the six analytical food categories, based on Meituan and Dianping data (December 2024).

In terms of accessibility, central communities (Clusters 2, 3, and 4) show higher accessibility in the W-FE than peripheral communities, especially for medium- and high-end dining categories such as Japanese cuisine, Jiangsu–Zhejiang cuisine, and Sichuan–Hunan cuisine. This pattern reflects a clear central–peripheral gradient. By contrast, accessibility is substantially higher in the OFDS-FE across all community types and food categories, consistent with H1. For example, in Cluster 0, the accessibility of rice noodles and wheat noodles increases from 9.87 to 147.75, representing an increase of more than 1500% (Figure 10). The improvement is particularly strong for affordable everyday dining, while medium- and high-end dining remains more dependent on central locations. Overall, accessibility follows a stable spatial gradient of central communities > non-central communities > suburban communities, indicating that locational conditions remain the dominant factor.

In terms of availability, the W-FE shows a consistent structure across community types, with affordable everyday dining being more available than medium- and high-end dining. The overall availability of the OFDS-FE is lower than that of the W-FE, but its internal differentiation is more evident. Affordable everyday dining is relatively more available in non-central and suburban communities, reaching approximately 50–80% of the corresponding W-FE level, while central communities show higher online availability for medium- and high-end dining. Therefore, the availability of medium- and high-end dining follows the order of central communities > non-central communities > suburban communities, whereas affordable everyday dining shows the opposite pattern.

In terms of affordability, central communities have lower affordability than peripheral communities in the W-FE, while differences associated with fee-and-age profiles within the same locational category are limited. In the OFDS-FE, affordability disparities across communities are reduced, but category-based differences remain. The affordability of affordable everyday dining declines slightly by approximately 0.2–12%, mainly due to delivery fees and other additional costs. In contrast, the affordability of medium- and high-end dining improves substantially. Japanese cuisine increases by more than 270% in Cluster 4, while Jiangsu–Zhejiang cuisine and Sichuan–Hunan cuisine generally increase by 50–100%. This suggests that the OFDS-FE is associated with higher relative affordability mainly for higher-end dining, while the change for affordable everyday dining is limited or slightly negative, supporting H3.

Overall, the W-FE shows a clear central–peripheral gradient: accessibility and availability follow the order of central communities > non-central communities > suburban communities, while affordability follows the opposite order. Compared with the W-FE, the OFDS-FE provides higher accessibility and affordability but lower availability. It also enlarges accessibility disparities, while reducing disparities in availability and affordability. Thus, OFDS is associated with expanded food access but does not fully eliminate the central–peripheral structure of the urban food environment.

### 5.2. Associations Between OFDS and the Equity of Restaurant-Service Access

Overall, the equity patterns associated with OFDS are dimension-specific rather than uniform. Inter-community disparities are smaller in the OFDS-FE most consistently for affordability, moderately for accessibility, and least consistently for availability. This suggests that OFDS is associated more strongly with expanded spatial choice and more equal price-related opportunities than with an improved balance between food supply and population demand. Cluster-level differences in indicator changes are statistically significant for all 18 dimension–category combinations (Kruskal–Wallis tests, all *p* < 0.01), and pairwise Dunn contrasts reported in Appendix E indicate which specific cluster differences are statistically supported.

**Figure 11:**
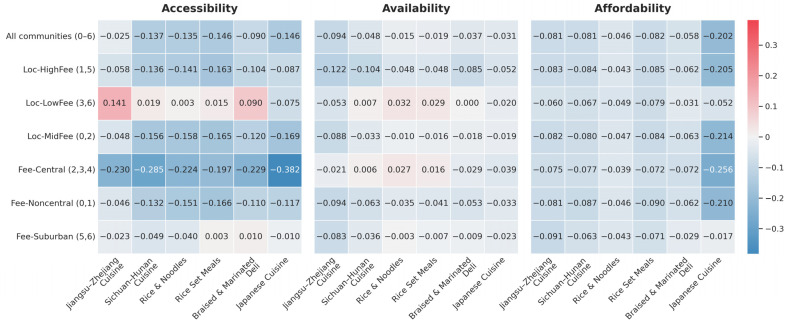
Heatmap of ΔG = Gini(OFDS-FE) − Gini(W-FE) by dimension, food category and community group (cluster definitions in Table 5; group definitions in Section 4.4). Negative values (blue) indicate smaller inter-community disparities in the OFDS-FE; positive values (red) indicate larger disparities. All values are based on modeled, supply-side indicators.

At the citywide level, disparities in food environment indicators are generally smaller in the OFDS-FE than in the W-FE, but the degree of change varies across dimensions and community groups (Figure 11). For all communities, ΔG ranges from −0.146 to −0.025 for accessibility, from −0.094 to −0.015 for availability, and from −0.202 to −0.046 for affordability. The strongest reduction appears in affordability, indicating that price-related disparities are substantially smaller in the OFDS-FE. However, this overall improvement masks clear heterogeneity across community types and food categories.

In the accessibility dimension, the largest decline in the Gini coefficient occurs in Fee-Central (Clusters 2, 3, and 4), where ΔG ranges from −0.367 to −0.194. However, in Loc-LowFee (Clusters 3 and 6), the Gini coefficient increases for some categories, with the highest increase reaching ΔG = 0.141. This indicates that expanded delivery ranges do not benefit all disadvantaged communities equally. Central old low-fee communities (Cluster 3) can gain more dining options through OFDS, while suburban older low-fee communities (Cluster 6) may still face limited access beyond the effective delivery range.

Availability shows a weaker and less consistent improvement. Although the Gini coefficient decreases in Loc-MidFee (Clusters 0 and 2), Loc-HighFee (Clusters 1 and 5), Fee-Noncentral (Clusters 0 and 1), and Fee-Suburban (Clusters 5 and 6), it increases slightly in Fee-Central (Clusters 2, 3, and 4) and Loc-LowFee (Clusters 3 and 6) for some affordable everyday dining categories, such as rice set meals and rice noodles and wheat noodles. Because mean availability is also lower in the OFDS-FE than in the W-FE for all six categories (Appendix F), the availability dimension is best characterized as equalization under shared scarcity—smaller disparities around a lower average level—rather than uniform improvement.

Affordability shows the most stable equity improvement. Across all community groups and food categories, ΔG is negative, with the largest reductions observed for Japanese cuisine and Jiangsu–Zhejiang cuisine. These categories were previously more expensive and spatially concentrated in central areas. By extending access to such higher-priced dining options, OFDS is associated with narrower spatial disparities in price-related access. However, this result should still be interpreted cautiously because the affordability measure, although including delivery fees, does not fully capture dynamic discounts, coupons, packaging fees, or time-varying platform pricing.

**Figure A3:**
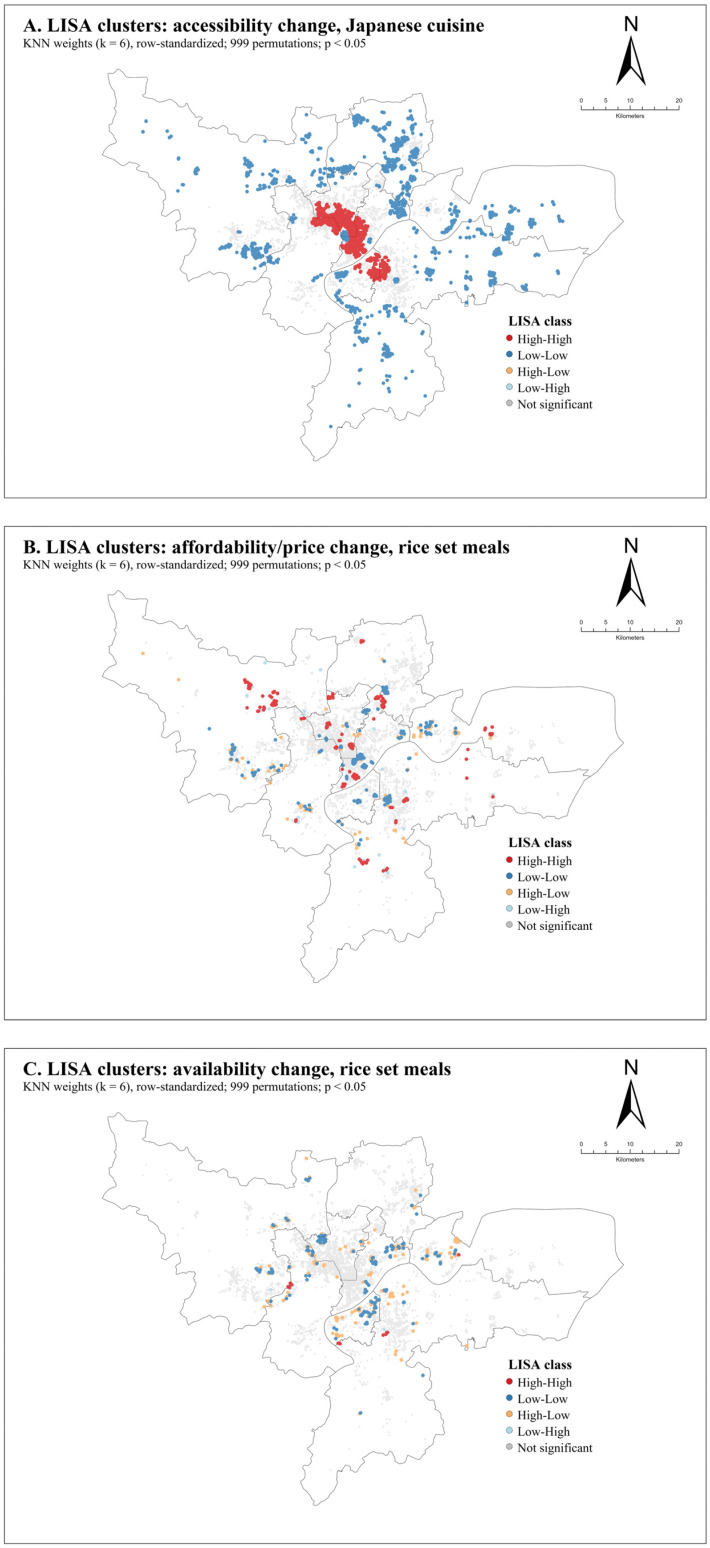
LISA cluster maps for representative changes associated with OFDS at the community scale. Panels show local indicators of spatial association for changes in accessibility, affordability/price and availability, calculated from community-level OFDS-FE and W-FE indicators. K-nearest-neighbour spatial weights (k = 6) were row-standardized, and significance was assessed using 999 permutations (*p* < 0.05).

The spatial structure of these changes is confirmed by global Moran’s I computed on representative indicators with k-nearest-neighbour weights (k = 6, row-standardized, 999 permutations; k = 8 as a robustness check): I ranges from 0.08 for the change in availability of rice set meals to 0.99 for accessibility levels and changes in the high-end category, with all permutation *p* = 0.001 (Appendix F, Table A7). Local indicators of spatial association (LISA) further show that high–high clusters of accessibility gains for the high-end category concentrate in central communities (mean distance to the city centre approximately 4.1 km), whereas low–low clusters lie in peripheral areas (approximately 14.7 km) (Appendix F, Figure A3); high–high clusters of affordability gains are more spatially diffuse and extend into non-central communities.

Taken together, OFDS is associated with a structured rather than homogeneous equalizing pattern: accessibility gains arise through delivery-based spatial extension; availability gains are weaker because supply remains constrained by population competition; and affordability disparities narrow most consistently because delivery expands access to previously centralized higher-priced dining categories. These results partially support H4: citywide inequality indicators decline in most dimensions, but the equalizing pattern is heterogeneous across dimensions, categories and community types, and the central–peripheral gradient persists. Thus, OFDS is associated with uneven changes in the equity of restaurant-service access across dimensions, food categories, and community types. These equity statements concern the spatial distribution of potential dining opportunities supplied through the platform, not realized utilization (Section 6.4).

## 6. Discussion

### 6.1. Platform-Mediated Reconfiguration of Food Access Conditions

The results suggest that OFDS is not associated with uniform improvement of the food environment; they support H1 and H3, support H2 in that availability changes are weaker, less consistent and less favorable than accessibility gains, and partially support H4. Instead, it reconfigures food access by reorganizing the costs of searching, travelling, waiting, and paying for food. In terms of accessibility, OFDS is associated with an expansion of residents’ choice sets beyond walkable catchments and with a reduction in the direct travel burden borne by consumers. However, this does not mean that distance constraints disappear. Rather, spatial distance is converted into delivery time, rider labour, and platform dispatching constraints. In terms of availability, the expansion of reachable restaurants does not necessarily translate into higher effective supply. Availability reflects not only the number of accessible restaurants, but also the relationship between restaurant supply and population demand. Therefore, even when OFDS enlarges the spatial range of food access, effective per capita availability may still be constrained by concentrated demand, limited restaurant capacity, and platform dispatching conditions. This explains why the improvement in availability is weaker and less consistent than that in accessibility. In terms of affordability, the effect of OFDS is also category-specific. For medium- and high-end dining, OFDS may improve relative affordability by enabling residents to access restaurants that were previously difficult to reach without bearing the full dine-in costs, such as travel time, on-site service, and dining ambience. By contrast, for affordable everyday meals, delivery fees and other additional charges may offset the advantage of low food prices. Thus, OFDS is more effective in equalizing access to higher-priced dining categories than in reducing the economic burden of daily food consumption.

Taken together, OFDS does not remove access costs, but redistributes them across consumers, restaurants, riders, and platform systems. Consumer travel is partly replaced by delivery labour, restaurant visibility is reorganized through digital interfaces, and waiting time is embedded into the coordinated process of food preparation, rider pickup, and delivery. This platform-mediated redistribution explains why accessibility gains are the most visible outcome of the OFDS-FE, while the associated patterns in availability and affordability remain uneven.

### 6.2. Central-Place Structure and Digital Central Service Zones

At the spatial-structural level, OFDS is associated with a restructuring of the urban food environment but does not eliminate the traditional central-place hierarchy. The OFDS-FE presents a dual pattern of basic service diffusion and higher-order service persistence. Affordable everyday dining becomes more widely accessible through delivery services, whereas medium- and high-end dining remains more strongly concentrated in urban core areas. In peripheral communities, access to higher-order dining is still limited, and the gap between central and peripheral areas may persist or even intensify in some categories.

This pattern indicates that OFDS is not a simple process of decentralization. Rather, it acts as a bounded extension of the existing central-place structure. In the terms of Section 2.2, delivery endogenizes the range variable of central place theory—replacing consumer travel with platform logistics—while threshold demand and the locations of supply remain unchanged; the digital central service zone is the spatial expression of this platform-produced range. Through the delivery system, platform-mediated services extend point-based physical food centers into digital central service zones with limited spatial coverage. Communities located within these zones can share more diverse dining resources from central areas, whereas communities beyond the effective delivery range still face a rapid decline in access.

Therefore, the digital food environment remains constrained by physical space. OFDS may weaken some intra-zone disparities, but it cannot fully overcome the structural limitations of offline restaurant distribution. For suburban communities with insufficient offline food service supply, platform delivery alone is unlikely to eliminate spatial disparities in the food environment.

These findings resonate with, and extend, evidence from other contexts. In Nanjing, online delivery was found to improve the equity of food accessibility overall while leaving suburban gaps [17], a pattern consistent with the bounded extension observed here. In England, the most deprived neighbourhoods exhibited the highest online food outlet access [18], whereas in Hangzhou central old low-fee communities (Cluster 3) benefit more than suburban older low-fee communities (Cluster 6); together these results suggest that whether platforms reach disadvantaged areas depends on their position relative to existing restaurant agglomerations rather than on deprivation per se. Evidence from Canada shows that delivery apps extend geographic reach while skewing towards less healthy options [16], and U.S.-based work documents the emergence of “cyber food swamps” [19]; the supply-side framework used here cannot speak to healthfulness, but the persistence of the central–peripheral gradient documented above suggests that platform expansion overlays, rather than replaces, existing retail hierarchies—a finding also reported for dining sustainability in Tokyo [29]. For planning systems outside China—such as 15-min-city policies in Europe or food-access interventions in North America—the implication is that delivery ranges can be incorporated as a supplementary access layer, but cannot substitute for physical food infrastructure in genuinely underserved peripheries. The mechanisms documented here are most likely to transfer to dense, high-penetration cities with mature delivery infrastructure; extrapolation to low-density, car-dependent settings should be cautious, and cross-city replication remains an important direction for future work.

### 6.3. Planning Implications for Community Food Services

The findings suggest that community life-circle planning should incorporate digital delivery services as a supplementary layer to conventional walking-based service areas. Current planning standards mainly emphasize “10–15-min life circles” and “5-min life circles” based on physical accessibility. However, the results show that OFDS can extend dining access beyond walkable catchments, especially in non-central and suburban communities. Future planning should therefore consider digital central service zones, while also recognizing their spatial limits.

Greater attention should also be paid to online–offline coordination. Existing planning evaluations often focus on offline facility density and per capita allocation, but these indicators may not fully capture platform-mediated food access. Future assessments could introduce indicators that measure the relationship between offline restaurant supply, OFDS coverage, delivery accessibility, and community demand. This would help identify areas where offline facilities appear sufficient but digital service access remains weak, or where platform coverage is wide but effective per capita availability remains limited.

Finally, differentiated planning strategies should be developed according to community type. Building on the cluster-specific findings, the following directions are proposed. For suburban older low-fee communities (Cluster 6), where the accessibility Gini coefficient rises for some categories because parts of these communities lie beyond effective delivery ranges, the priority remains physical supply: reserving space for affordable everyday dining and community canteens in life-circle upgrading, before—rather than instead of—relying on delivery. On the platform side, municipalities could negotiate designated delivery-extension zones in which dispatch incentives are adjusted and delivery fees or minimum-order thresholds are capped for underserved communities, so that the platform’s effective service boundary is treated as a plannable object rather than an unregulated outcome. For suburban newer high-fee communities (Cluster 5), modeled OFDS coverage could be incorporated into the commercial-allocation review of new suburban sub-centres: where delivery accessibility is already high, planned catering floorspace can prioritize formats that delivery does not substitute, such as group dining and fresh-food retail; where it is low, reserved catering space builds the offline base that delivery can subsequently extend. For central older communities (Clusters 2–4), which already lie inside dense digital central service zones, the main tasks are managing the externalities of high-intensity delivery—designated rider pick-up points and micro-hubs, curb-side management around restaurant agglomerations, and building-entry protocols in old residential compounds—and supporting the digital participation of legacy small eateries and community or institutional canteens through onboarding assistance and commission-relief pilots, so that affordable everyday options remain represented online. At the governance level, community life-circle assessment could add a delivery coverage layer alongside conventional offline indicators: decay-weighted delivery accessibility, per-capita online availability and delivered-cost affordability of the kind measured in this study, computed from platform service-area data shared with planning authorities at the district level. Such indicators would allow planners to identify communities where offline provision appears adequate but effective platform access is weak, or vice versa, and would give platform-regulation instruments—service-area transparency and fee caps in designated underserved zones—an evidence base. These recommendations remain preliminary and model-dependent: they rest on supply-side, single-platform, single-period evidence, and are proposed as directions for assessment and negotiation rather than as prescriptive standards.

### 6.4. Limitations

Several limitations should be noted. First, the online food environment is represented by records from a single platform, Meituan. Meituan is the leading OFDS platform in China—recent market reporting citing BOCOM International estimated that it accounted for approximately 65% of China’s food-delivery market by transaction share in 2024, compared with approximately 33% for Ele.me and 2% for other platforms [45]—but its listing, ranking and merchant-participation rules may differ from those of other platforms. The results should therefore be read as evidence from the leading platform rather than as a complete census of platform-mediated food delivery. The findings should accordingly be interpreted as characterizing the food environment within the Meituan platform ecosystem—the largest, but not the only, food-delivery ecosystem in China—rather than the overall food environment of Hangzhou or of Chinese cities in general. Restaurants that are not listed on Meituan, restaurants operating exclusively on other platforms such as Ele.me, and outlets operating under different business models (for example, direct telephone- or WeChat-based ordering with in-house delivery) are not represented on the OFDS side of the comparison. Two further data boundaries follow: multi-brand virtual storefronts operating from a single licensed kitchen cannot be distinguished in the records and may inflate nominal online restaurant counts, and some residual undercoverage remains from the unmatched records excluded in Section 3.2.2. Future research should compare Meituan, Ele.me and other platforms when comparable restaurant-level data become accessible.

Second, the study measures potential access from the supply side rather than realized consumer behavior. The OFDS-FE indicators describe the set of restaurants that could be reached through platform delivery under the observed delivery-time and distance-decay conditions; they do not capture individual orders, workplace exposure, daily activity spaces, household preferences or platform recommendation algorithms. Because these factors shape realized dietary choices, they should be incorporated once individual mobility or transaction data become available. This boundary has direct implications for the equity findings. Realized utilization additionally depends on consumer preferences, income and food-budget shares, digital literacy, ordering frequency, age structure and platform-interaction skills, all of which may vary systematically across communities. Because groups with lower digital literacy or a lower propensity to order online—plausibly over-represented in older and low-fee communities—may convert less of the measured potential access into actual use, the equalization of potential access reported here may overstate the equalization of realized access for precisely those communities. The equity conclusions of this study should therefore be read as statements about the spatial distribution of dining opportunities supplied through the platform, not about the distribution of realized food consumption or dietary outcomes; linking the two requires individual-level order, mobility or survey data, which we identify as the priority for future research.

Third, the analysis relies on a single-period cross-section and on community-level proxy variables. Building age, distance to the city center and property management fee capture salient dimensions of urban location and housing-market stratification, but they are no substitute for direct measures of income, age structure, migrant status or household composition. The community typology should accordingly be read as an interpretive stratification rather than a causal classification of socio-demographic groups.

Fourth, Euclidean buffers overstate walkable access in the W-FE wherever street networks impose detours. The network-distance analysis on a stratified subsample (Appendix H) yields a median detour ratio of 1.51 (IQR 1.36–1.81), with network-based W-FE accessibility at 33.6–42.9% of the Euclidean level; community and cluster orderings, however, are strongly preserved (Spearman ρ = 0.825–0.920 by category and 0.972 for cluster–category means). Main-text W-FE levels should therefore be read as upper bounds, and because the OFDS-FE already embeds delivery-time constraints, the reported W-FE/OFDS-FE contrasts are conservative with respect to network detours.

Finally, the core comparison rests on six restaurant categories, which together account for roughly half of the restaurant pool and of recorded monthly OFDS sales (Section 3.2.3). The excluded categories are mostly seasonal, occasion-specific or internally heterogeneous, and their food environments may follow different spatial logics; the findings therefore speak to everyday staple and mainstream full-service dining rather than to the entire restaurant market. Relatedly, the E2SFCA availability measure assumes identical supply capacity across restaurants, because no capacity indicator is observed comparably for offline and online restaurants (Section 4.2.3). Availability results thus reflect the spatial configuration of supply relative to demand rather than differences in individual restaurant capacity, and capacity weighting should be revisited when comparable seating, floor-area or order-capacity data become available.

## 7. Conclusions

This study develops an integrated online–offline analytical framework to evaluate how OFDS is associated with the restructuring of the urban food environment in Hangzhou. The findings suggest that OFDS-FE extends the spatial range of dining access beyond walkable catchments, particularly in non-central and suburban communities. However, this extension does not produce uniform improvements across all dimensions. Compared with the W-FE, the OFDS-FE is associated with higher accessibility and more equal affordability for medium- and high-end dining categories, while improvements in the availability of affordable everyday dining remain relatively limited due to population competition, delivery fees, and other additional costs. Overall, OFDS does not dissolve the traditional central-place structure of urban food services. Higher-order dining resources remain largely anchored in urban core areas. Taken as a whole, the evidence supports H1–H3 and partially supports H4. All of these findings concern supply-side potential access within the Meituan platform ecosystem, rather than realized consumer utilization.

Because OFDS depends on offline restaurant networks as its supply base, its service radius remains constrained by physical space. Therefore, OFDS should not be understood as a replacement for the traditional central-place structure, but rather as a bounded extension of offline food service provision. It may extend the service scope of physical centers into digital central service zones, within which residents can access a wider range of restaurant options through delivery services. Beyond these zones, however, the benefits of OFDS decline rapidly. In this sense, OFDS may weaken some intra-zone disparities, but it does not fundamentally break the concentration of higher-order dining services in urban cores, nor can it fully replace the role of offline dine-in services in urban food access.

This study makes three main contributions. First, it develops an integrated measurement framework for comparing the W-FE and the OFDS-FE at the same community scale and across the same food categories. By incorporating accessibility, availability, and affordability, the framework moves beyond a single focus on physical distance and provides a more comprehensive basis for assessing online–offline food environment differences. Second, it constructs a delivery-process-based segmented distance decay function for the OFDS-FE. This approach avoids the simplification of using a fixed delivery radius and better approximates the decline in platform service intensity with increasing delivery distance. Third, from the perspective of central place theory, this study proposes the concept of the digital central service zone. This concept helps explain why OFDS may extend the service reach of existing food centers without fully eliminating the central–peripheral hierarchy of urban food services.

The findings also have practical implications for community life-circle planning. Digital delivery ranges should be considered as a supplementary layer to conventional walking-based service areas, especially when identifying underserved communities and evaluating online–offline food service coordination. At the same time, planning practice should not overestimate the equalizing role of OFDS. Since platform-based food access remains dependent on offline restaurant distribution, delivery capacity, and platform mechanisms, policymakers and platform companies should pay attention to potential new forms of food access inequality and guide platform resources toward communities with weaker food service provision.

## Figures and Tables

**Table 1:** Data Sources and Key Attributes.

Data Source Name	Platform/Source	Collection Period	Key Attributes
OFDS Restaurant Data	Meituan	December 2024	Restaurant name, address, coordinates, cuisine type, monthly sales, avg. price per person, delivery fee, delivery time, etc.
On-site Dining Restaurant Data	Dianping	December 2024	Restaurant name, address, coordinates, cuisine type, avg. price per person.
Community Data	Anjuke	2024	Community name, address, year built, total households, floor area ratio, property fee, avg. unit price, etc.

**Table 2:** Restaurant Classification and Selection Criteria for the Core Comparative Analysis.

Broad Group	Subcategory	Offline Avg. Price (CNY/Person)	Offline N	OFDS Avg. Price (CNY/Person)	OFDS N	Included	Reason for Inclusion/Exclusion
Full-service	Jiangsu–Zhejiang cuisine	84.5	3731	32.6	1081	Yes	Local full-service cuisine; sufficient records
	Sichuan–Hunan cuisine	51.8	2136	25.4	1581	Yes	Common full-service cuisine; high offline and online presence
	Japanese cuisine	178.5	618	41.2	292	Yes	High-end dining category; clear price gradient
	Fujian–Cantonese cuisine	98.5	553	30.1	232	No	Relatively limited OFDS observations
	Other regional cuisines	54.5	3896	27.2	2533	No	Internally heterogeneous category
	Private dining	133.2	1062	45	36	No	Very limited OFDS records
	Seafood &freshwater cuisine	149.2	658	51.9	309	No	Strong freshness and dine-in dependence
	Western food	102.2	737	31.1	216	No	Limited OFDS observations and mixed formats
	Southeast Asian cuisine	76.2	129	24.3	132	No	Low sample size
	Korean cuisine	69.2	380	22.2	195	No	Relatively limited coverage
	Sichuan hotpot	77.6	774	40.1	259	No	Strong group-dining and experiential attributes
	Beef and mutton hotpot	76.1	728	62	79	No	Very limited OFDS records
	Other hotpot	64.3	892	29.2	486	No	Strong dine-in and social dining attributes
	Barbecue	64.1	1697	31.5	1508	No	Time-specific and experiential consumption
	Crayfish and grilled fish	71.3	1019	48.2	445	No	Seasonal and group-dining characteristics
	Grilled meat	92.5	1041	—	—	No	No comparable OFDS records
Fast-food/snack	Rice noodles and wheat noodles	22.1	6431	18.5	4510	Yes	High-frequency staple food; large sample size
	Rice set meals	23.8	11,421	18.1	4748	Yes	Standardized everyday meal; largest sample size
	Dumplings and wontons	23.7	1212	18.3	1004	No	Similar to staple fast-food categories
	Western fast food	26.4	1897	24.3	2534	No	Chain-dominated and highly standardized
	Porridge and steamed buns	15	1572	15.6	1565	No	Breakfast-oriented and time-specific demand
	Specialty snacks	29.9	2143	20.1	2530	No	Internally heterogeneous snack category
	Street snacks	19.9	2216	15.8	1468	No	Snack-oriented and less comparable as full meals
	Fried snacks	23.4	1229	30.8	279	No	Limited OFDS records and snack-oriented category
	Braised and marinated deli foods	28.5	1853	32.7	1619	Yes	Ready-to-eat food; comparable offline and online forms

**Table 3:** Definitions and Measurement of Three Food Environment Dimensions.

Dimension	Metric	Definition	Characterization
Spatial access	Accessibility	The spatial ease with which residents can reach food service resources within a defined service range.	Reflects the range and richness of dining choices available to residents after accounting for distance decay.
Supply–demand	Availability	The relative match between restaurant supply and community population demand.	Reflects the degree of supply–demand pressure or potential service congestion within the food environment.
Economic cost	Affordability	Residents’ relative purchasing capacity for accessing specific food categories.	Reflects the relative economic burden of obtaining food services across communities.

**Table 4:** Definitions and Calculation of Food Environment Indicators.

Food Environment	Metric	Spatial Scope	Calculation Formula
W-FE	Accessibility	full-service restaurants 1.5 km fast-food restaurants 800 m	${Accessibility}_{i}^{W-FE}=\sum_{c\in \varepsilon (d_{ic}\leq d_{0}^{W-FE})}G_{W-FE}(d_{ic})$
	Availability		STEP 1: $R_{c}^{W-FE}=\frac{S_{C}}{\sum_{k\in \varepsilon (d_{ic}\leq d_{0}^{W-FE})}G_{W-FE}(d_{kc})P_{k}}$ STEP 2: ${Availability}_{i}^{W-FE}=\sum_{c\in \varepsilon (d_{ic}\leq d_{0}^{W-FE})}G_{W-FE}(d_{ic})R_{c}^{W-FE}$
	Affordability		${Affordability}_{i}^{W-FE}=\frac{\sum_{c\in \varepsilon (d_{ic}\leq d_{0}^{W-FE})}G_{W-FE}(d_{ic})Y}{\sum_{c\in \varepsilon (d_{ic}\leq d_{0}^{W-FE})}G_{W-FE}(d_{ic}){\bar{p}}_{c}}$ *${\bar{p}}_{c}$ = ${\bar{p}}_{food}$
OFDS-FE	Accessibility	8 km Max Service Range	${Accessibility}_{i}^{OFDS-FE}=\sum_{c\in \varepsilon (d_{ic}\leq d_{0}^{OFDS-FE})}G_{OFDS-FE}(d_{ic})$
	Availability		STEP 1: $R_{c}^{OFDS-FE}=\frac{S_{C}}{\sum_{k\in \varepsilon (d_{ic}\leq d_{0}^{OFDS-FE})}G_{OFDS-FE}(d_{kc})P_{k}}$ STEP 2: ${Availability}_{i}^{OFDS-FE}=\sum_{c\in \varepsilon (d_{ic}\leq d_{0}^{OFDS-FE})}G_{OFDS-FE}(d_{ic})R_{c}^{OFDS-FE}$
	Affordability		${Affordability}_{i}^{OFDS-FE}=\frac{\sum_{c\in \varepsilon (d_{ic}\leq d_{0}^{OFDS-FE})}G_{OFDS-FE}(d_{ic})Y}{\sum_{c\in \varepsilon (d_{ic}\leq d_{0}^{OFDS-FE})}G_{OFDS-FE}(d_{ic}){\bar{p}}_{c}}$ *${\bar{p}}_{c}$ *=* ${\bar{p}}_{food}$ + ${\bar{p}}_{delivery}$

Variable key: *i*: Community, *c*: Restaurant, *Pk*: Population of unit, *pc*: Average per capita price of restaurant *c*, *Y*: Average daily expenditure of residents, *Sc*: Supply capacity of restaurant *c*, *d*0: Maximum service distance buffer, G(dic): Distance decay function.

**Table 5:** Descriptive Statistics and Unified Definitions of Community Clusters.

Cluster	Count (N)	Property Fee (CNY/m^2^)		Distance to City Center (km)		Community Age (Years)		Cluster Definition
		Mean	Median	Mean	Median	Mean	Median	
0	1769	0.9	1.0	12.2	12.4	16.2	15.1	Non-central older low-fee communities
1	1297	3.0	3.1	10.3	10.2	8.4	8.2	Non-central newer high-fee communities
2	629	1.8	1.7	4.3	4.3	20.2	20.1	Central older mid-fee communities
3	111	0.3	0.4	3.4	3.5	25.3	24.3	Central old low-fee communities
4	164	0.8	0.8	3.3	3.4	33.4	35.6	Central oldest communities
5	278	2.0	2.1	20.3	20.5	10.4	10.5	Suburban newer high-fee communities
6	397	0.7	0.8	24.5	25.0	17.2	18.2	Suburban older low-fee communities
Total/Mean	4645	1.8	1.5	15.4	14.2	18.2	21	

**Figure A2:**
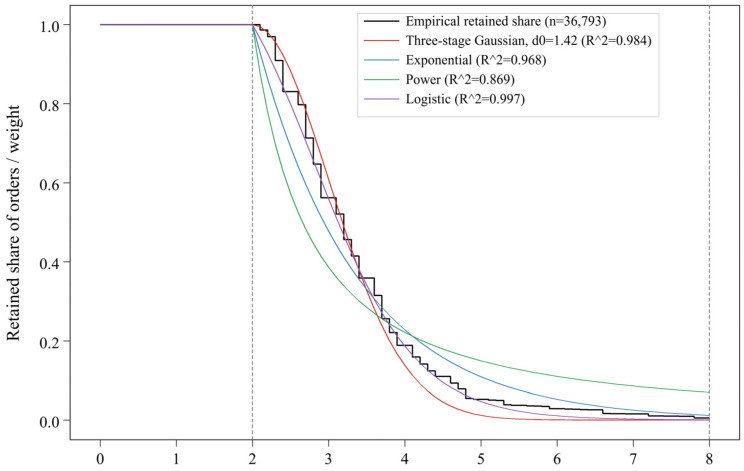
Empirical retained-order curve of OFDS orders (*n* = 36,793, Meituan, December 2024) and fitted three-stage Gaussian, exponential, power and logistic decay functions. The retained-order curve is normalized to 1 at 2 km; the vertical axis is the retained share of orders or fitted weight, and the horizontal axis is the implied delivery distance (km).

## Data Availability

The data presented in this study are available on request from the corresponding author. The OFDS and POI data were obtained from third-party platforms and are subject to their terms of use.

## Appendix A. Enhanced Two-Step Floating Catchment Area (E2SFCA) Method

### Appendix A.1

Step 1: For each restaurant c (supply point), a catchment area is established based on the maximum service range $d_{0}$. All population $P_{k}$ within this catchment area is summed, weighting each population unit by the distance decay function $G(d_{ic})$. This calculates the supply-to-demand ratio $R_{c}$ for restaurant c.

(A1) $$R_{c}=\frac{Sc}{\sum_{k\in \{d_{kc}\leq d_{0}\}}G(d_{ic})P_{k}}$$

where $P_{k}$ is the population of community k, $d_{kc}$ is the distance between k and c (where $d_{kc}\leq d_{0}$), Sc is the supply capacity (assumed to be identical for all restaurants), and $G(d_{ic})$ is the specific distance decay function addressing spatial friction.

Step 2: For each community i (demand point), a catchment area is established using the same service range $d_{0}$. The Availability for community i is calculated by summing the supply-to-demand ratios $R_{c}$ of all restaurants c within its catchment, again weighted by the distance decay function $G(d_{ic})$.

(A2) $$A_{i}^{D}=\sum_{k\in \{d_{ic}\leq d_{0}\}}G(d_{ic})R_{c}$$

### Appendix A.2

The Gini coefficient (*G*) is calculated as follows:

(A3) $$G=\frac{\Sigma_{i=1}^{n}\Sigma_{j=1}^{n}|x_{i}-x_{j}|}{2n^{2}\bar{x}\;}$$

where *xi* is the food environment metric value (e.g., Accessibility or Affordability) for community *i*, and *x* is the mean value of that metric across all *n* communities. The coefficient ranges from 0 to 1. As *G* → 0, the distribution of resources among communities is more even, indicating higher spatial equity. As *G* → 1, disparities are more significant, indicating stronger spatial inequality.

## Appendix B. Cross-Platform Matching Validation

Appendix B reports the manual validation of the Meituan-Dianping matching procedure. We drew 500 stratified matched pairs from the six analytical food categories and checked each pair against restaurant name, brand, category, address and coordinate proximity. Of these pairs, 498 were confirmed as true matches and two remained ambiguous after manual review. Treating the ambiguous cases as non-confirmed yields a conservative precision estimate of 99.6%. We also examined 200 sampled unmatched Meituan records; all were judged to have no corresponding Dianping record in the validation sample. Finally, Figure A1 maps the unmatched Meituan records and shows that they are distributed across the main urban area and nearby suburban commercial nodes rather than concentrated in a single omitted zone. These validation results support the reliability of the matching procedure while motivating the undercoverage caveat in Section 6.4.

## Appendix C. Sensitivity of Delivery-Model Parameters

Appendix C examines how the OFDS distance-decay model responds to two behavioural assumptions in Section 4.2.2: the non-riding waiting time *T2* and the average rider speed *v*. The recalculation uses the Meituan delivery-duration sample (*n* = 36,793 orders). Because delivery duration and waiting time are measured in minutes while rider speed is measured in km/h, the implied restaurant-to-consumer delivery distance is calculated as *D* = max($T_{total}$ − $T_{2}$, 0) × *v*/120. Negative implied distances, which occur only for a small number of short-duration orders under the $T_{2}$ = 20 min scenario, are set to zero.

The empirical retained-order curve is defined as P(D ≥ d | D ≥ 2 km) over d = 2–8 km, so that the curve is normalized to 1 at the beginning of the modeled decay segment. The three-stage Gaussian decay function is then fitted to this empirical curve. Table A1 reports the refitted parameter d0, goodness-of-fit statistics, the shares of implied delivery distances within 5 km and 8 km, and the half-decay distance, defined as 2 + d0 × √ln 2.

**Table A1:** Refitted decay parameters across the waiting-time × rider-speed grid (*n* = 36,793 orders).

T2 (min)	v (km/h)	d0 (km)	R^2^	RMSE	MAE	≤5 km (%)	≤8 km (%)	Half-Decay (km)
5	10	0.75	0.958	0.0484	0.0304	98.5	100.0	2.6
5	12	1.27	0.994	0.0237	0.0187	96.7	100.0	3.1
5	15	2.05	0.995	0.0257	0.0200	89.5	99.1	3.7
5	18	2.91	0.981	0.0527	0.0397	70.0	97.4	4.4
10	10	0.61	0.934	0.0552	0.0340	99.1	100.0	2.5
10	12	0.91	0.965	0.0480	0.0333	97.5	100.0	2.8
10	15	1.42	0.983	0.0387	0.0322	95.2	99.5	3.2
10	18	2.09	0.996	0.0227	0.0194	86.5	98.5	3.7
15	10	0.49	0.846	0.0747	0.0433	99.6	100.0	2.4
15	12	0.72	0.932	0.0594	0.0410	98.5	100.0	2.6
15	15	0.99	0.925	0.0674	0.0498	96.7	99.9	2.8
15	18	1.47	0.966	0.0539	0.0450	94.8	99.0	3.2
20	10	0.55	0.793	0.0920	0.0544	100.0	100.0	2.5
20	12	0.68	0.883	0.0746	0.0496	99.1	100.0	2.6
20	15	0.82	0.892	0.0758	0.0564	97.5	100.0	2.7
20	18	1.21	0.946	0.0633	0.0541	96.5	99.5	3.0

Under the adopted setting in the main analysis ($T_{2}$ = 10 min, v = 15 km/h), the refitted decay parameter is d0 = 1.42 km, with R^2^ = 0.983. The corresponding half-decay distance is approximately 3.2 km, and 99.5% of implied delivery distances fall within the 8 km service ceiling. Across the full sensitivity grid, at least 97.4% of implied delivery distances remain within 8 km, indicating that the 8 km ceiling is not a binding constraint for the observed order-duration distribution. The fitted model performs best around lower-waiting and moderate-to-high-speed scenarios. Fit deteriorates when T2 is set to 20 min because a larger share of observed orders then falls at or below the 2 km plateau.

## Appendix D. Alternative Distance-Decay Specifications

Appendix D (Table A2 and Figure A2) compares the adopted three-stage Gaussian decay function with three alternative families fitted to the same normalized empirical retained-order curve under the adopted setting ($T_{2}$ = 10 min, v = 15 km/h).

**Table A2:** Goodness of fit of alternative decay families on the normalized empirical retained-order curve.

Function	Parameter(s)	R^2^	RMSE	MAE
Three-stage Gaussian	d0 = 1.42	0.9835	0.0387	0.0322
Exponential	λ = 1.35	0.9679	0.0540	0.0414
Power	α = 1.37	0.8685	0.1093	0.0942
Logistic	μ = 2.83; s = 0.66	0.9970	0.0166	0.0135

The logistic function provides the closest numerical fit, while the three-stage Gaussian also fits the empirical retained-order curve well. The three-stage Gaussian is retained for two reasons. First, it is consistent with the Gaussian decay structure used for the W-FE, which helps maintain comparability between the walkable and delivery-based food environments. Second, its 0–2 km plateau has a substantive interpretation: at short distances, platform-recorded delivery time is dominated by fixed waiting and handover components rather than riding distance alone. The three-stage Gaussian function therefore provides a theoretically interpretable and empirically adequate approximation of OFDS distance decay.

## Appendix E. Statistical Tests of Environment and Cluster Differences

Appendix E reports the statistical evidence underlying the comparative statements in Section 5. Paired Wilcoxon signed-rank tests compare the W-FE and OFDS-FE values of each indicator within communities for each of the 18 dimension–category combinations, with Benjamini–Hochberg correction for multiple testing; effect sizes are reported as r = |Z|/√n (Table A3). All 18 contrasts are significant at adjusted *p* < 0.001. Accessibility is uniformly higher in the OFDS-FE (r = 0.84–0.87), availability is uniformly lower (r = 0.22–0.64), and affordability is higher in the OFDS-FE for the three full-service categories (r = 0.82–0.86) but lower for the three everyday categories (r = 0.33–0.58), in which delivery fees offset low food prices—consistent with H2 and H3.

**Table A3:** Paired Wilcoxon signed-rank tests, W-FE versus OFDS-FE, by dimension and category. Medians are community-level indicator values; *p*-values are Benjamini–Hochberg adjusted.

Dimension	Category	n Pairs	Median W-FE	Median OFDS-FE	Effect Size r	Adj. *p*	Direction
Accessibility	Jiangsu–Zhejiang cuisine	4487	8.45	43.60	0.855	<0.001	OFDS-FE > W-FE
Accessibility	Sichuan–Hunan cuisine	4481	8.47	64.10	0.866	<0.001	OFDS-FE > W-FE
Accessibility	Rice noodles and wheat noodles	4535	8.84	165.90	0.866	<0.001	OFDS-FE > W-FE
Accessibility	Rice set meals	4561	15.08	176.27	0.866	<0.001	OFDS-FE > W-FE
Accessibility	Braised and marinated deli foods	4229	3.35	65.23	0.866	<0.001	OFDS-FE > W-FE
Accessibility	Japanese cuisine	3737	3.36	18.22	0.843	<0.001	OFDS-FE > W-FE
Availability	Jiangsu–Zhejiang cuisine	4487	0.20	0.15	0.456	<0.001	OFDS-FE < W-FE
Availability	Sichuan–Hunan cuisine	4481	0.20	0.19	0.252	<0.001	OFDS-FE < W-FE
Availability	Rice noodles and wheat noodles	4535	0.65	0.53	0.304	<0.001	OFDS-FE < W-FE
Availability	Rice set meals	4561	1.07	0.54	0.640	<0.001	OFDS-FE < W-FE
Availability	Braised and marinated deli foods	4229	0.20	0.20	0.221	<0.001	OFDS-FE < W-FE
Availability	Japanese cuisine	3737	0.07	0.04	0.555	<0.001	OFDS-FE < W-FE
Affordability	Jiangsu–Zhejiang cuisine	4487	2.67	3.87	0.825	<0.001	OFDS-FE > W-FE
Affordability	Sichuan–Hunan cuisine	4481	2.67	4.73	0.855	<0.001	OFDS-FE > W-FE
Affordability	Rice noodles and wheat noodles	4535	6.07	5.60	0.577	<0.001	OFDS-FE < W-FE
Affordability	Rice set meals	4555	6.01	5.71	0.332	<0.001	OFDS-FE < W-FE
Affordability	Braised and marinated deli foods	4229	4.81	3.78	0.815	<0.001	OFDS-FE < W-FE
Affordability	Japanese cuisine	3730	1.19	2.81	0.826	<0.001	OFDS-FE > W-FE

Kruskal–Wallis tests examine whether indicator changes differ across the seven community clusters (Table A4). All 18 tests are significant (*p* < 0.01); effect sizes (ε^2^) are large for accessibility (0.17–0.42) and modest for availability (0.004–0.045) and affordability (0.006–0.064), indicating that cluster membership structures accessibility gains much more strongly than the other two dimensions. Post-hoc pairwise Dunn tests with Benjamini–Hochberg correction (378 contrasts, summarized as a compact letter display in Table A5) show that 237 of the 378 pairwise cluster differences are significant. Non-significant contrasts concentrate in availability (81 of 126) and, to a lesser extent, affordability (51 of 126), whereas accessibility contrasts are almost uniformly significant (117 of 126)—a pattern that itself corroborates the characterization of availability changes as weaker and less consistent (H2). Because community indicators are spatially autocorrelated (Appendix F), the tests in this appendix should be read as strong descriptive evidence rather than exact inference under independence, and interpretation in the main text rests primarily on the reported effect sizes.

**Table A4:** Kruskal–Wallis tests of indicator changes (Δ = OFDS-FE − W-FE) across the seven community clusters (df = 6).

Dimension	Category	n	H	ε^2^	*p*
Accessibility	Jiangsu–Zhejiang cuisine	4487	1882.9	0.419	<0.001
Accessibility	Sichuan–Hunan cuisine	4481	1005.1	0.223	<0.001
Accessibility	Rice noodles and wheat noodles	4535	1157.9	0.254	<0.001
Accessibility	Rice set meals	4561	786.7	0.171	<0.001
Accessibility	Braised and marinated deli foods	4229	1419.7	0.335	<0.001
Accessibility	Japanese cuisine	3737	800.7	0.213	<0.001
Availability	Jiangsu–Zhejiang cuisine	4487	208.8	0.045	<0.001
Availability	Sichuan–Hunan cuisine	4481	30.7	0.006	<0.001
Availability	Rice noodles and wheat noodles	4535	35.7	0.007	<0.001
Availability	Rice set meals	4561	44.2	0.008	<0.001
Availability	Braised and marinated deli foods	4229	22.3	0.004	0.0011
Availability	Japanese cuisine	3737	29.1	0.006	<0.001
Affordability	Jiangsu–Zhejiang cuisine	4487	62.3	0.013	<0.001
Affordability	Sichuan–Hunan cuisine	4481	98.0	0.021	<0.001
Affordability	Rice noodles and wheat noodles	4535	294.3	0.064	<0.001
Affordability	Rice set meals	4555	33.5	0.006	<0.001
Affordability	Braised and marinated deli foods	4229	45.7	0.009	<0.001
Affordability	Japanese cuisine	3730	126.8	0.032	<0.001

**Table A5:** Compact letter display of the pairwise Dunn tests of indicator changes (Δ = OFDS-FE − W-FE) across the seven community clusters (Benjamini–Hochberg adjusted, α = 0.05; 378 contrasts). Within each row, clusters sharing a letter do not differ significantly, and clusters with no letter in common differ significantly; letters are assigned in descending order of the cluster-median change. The full contrast-level table is available from the corresponding author on request.

Dimension	Category	Cluster 0	Cluster 1	Cluster 2	Cluster 3	Cluster 4	Cluster 5	Cluster 6
Accessibility	Jiangsu-Zhejiang cuisine	d	c	b	a	a	e	f
Accessibility	Sichuan-Hunan cuisine	d	c	b	a	ab	e	f
Accessibility	Japanese cuisine	d	c	b	a	b	e	e
Accessibility	Rice noodles and wheat noodles	d	c	b	a	a	e	f
Accessibility	Rice set meals	d	c	b	a	ab	e	f
Accessibility	Braised and marinated deli foods	d	c	b	a	a	e	f
Availability	Jiangsu-Zhejiang cuisine	b	b	a	a	a	b	b
Availability	Sichuan-Hunan cuisine	bc	c	ab	abc	abc	a	ab
Availability	Japanese cuisine	a	b	ab	ab	b	ab	a
Availability	Rice noodles and wheat noodles	c	bcd	bcd	d	bd	bc	a
Availability	Rice set meals	bd	ce	cd	e	cde	a	ab
Availability	Braised and marinated deli foods	a	a	b	ab	ab	a	a
Affordability	Jiangsu-Zhejiang cuisine	c	c	b	bc	a	b	bc
Affordability	Sichuan-Hunan cuisine	b	b	b	b	a	b	c
Affordability	Japanese cuisine	c	b	d	e	bc	bcd	a
Affordability	Rice noodles and wheat noodles	d	c	b	ab	a	e	e
Affordability	Rice set meals	a	a	a	a	a	a	b
Affordability	Braised and marinated deli foods	c	c	b	a	ab	cd	d

## Appendix F. Inequality Indicators and Spatial Clustering

Appendix F reports the citywide Gini coefficients of both environments together with the corresponding mean levels (Table A6), so that changes in inequality can be interpreted jointly with changes in average levels rather than in isolation. For accessibility and for the affordability of full-service categories, the OFDS-FE combines lower Gini coefficients with higher means—narrower disparities around a higher level. For availability, and for the affordability of the three everyday categories, the Gini coefficient falls while the mean also falls: these dimensions exhibit equalization under shared scarcity, and the corresponding statements in Section 5.2 and Section 6 are worded accordingly. Cluster-by-category means and medians are reported in Table A8; full P10–P90 percentile ranges are available from the corresponding author on request.

**Table A6:** Citywide Gini coefficients and mean indicator levels, W-FE versus OFDS-FE, by dimension and category. ΔG = Gini(OFDS-FE) − Gini(W-FE).

Dimension	Category	Gini W-FE	Gini OFDS-FE	ΔG	Mean W-FE	Mean OFDS-FE
Accessibility	Jiangsu–Zhejiang cuisine	0.430	0.405	−0.025	10.74	50.76
Accessibility	Sichuan–Hunan cuisine	0.430	0.294	−0.137	10.74	57.12
Accessibility	Rice noodles and wheat noodles	0.411	0.276	−0.134	10.77	154.97
Accessibility	Rice set meals	0.424	0.279	−0.146	18.52	161.26
Accessibility	Braised and marinated deli foods	0.398	0.308	−0.090	4.00	60.25
Accessibility	Japanese cuisine	0.591	0.445	−0.146	6.18	17.52
Availability	Jiangsu–Zhejiang cuisine	0.650	0.556	−0.094	0.47	0.23
Availability	Sichuan–Hunan cuisine	0.650	0.603	−0.048	0.47	0.34
Availability	Rice noodles and wheat noodles	0.611	0.596	−0.015	1.32	0.97
Availability	Rice set meals	0.631	0.611	−0.019	2.32	1.03
Availability	Braised and marinated deli foods	0.617	0.581	−0.037	0.43	0.35
Availability	Japanese cuisine	0.650	0.619	−0.031	0.16	0.07
Affordability	Jiangsu–Zhejiang cuisine	0.113	0.038	−0.075	2.74	3.92
Affordability	Sichuan–Hunan cuisine	0.113	0.037	−0.076	2.74	4.72
Affordability	Rice noodles and wheat noodles	0.073	0.030	−0.044	6.10	5.60
Affordability	Rice set meals	0.109	0.031	−0.078	6.14	5.69
Affordability	Braised and marinated deli foods	0.090	0.032	−0.058	4.85	3.81
Affordability	Japanese cuisine	0.318	0.151	−0.167	1.43	3.25

Global Moran’s I statistics computed on representative level and change indicators with k-nearest-neighbour spatial weights (k = 6, row-standardized, projected coordinates, 999 permutations; k = 8 as a robustness check) are reported in Table A7. All statistics are significant (permutation *p* = 0.001). Accessibility levels and changes are very strongly spatially clustered (I = 0.77–0.99), affordability changes moderately so (I = 0.48–0.51), and availability changes only weakly (I = 0.08). Figure A3 maps the LISA classifications for representative changes and shows that high-high clusters of accessibility gains for the high-end category concentrate in central communities, whereas low-low clusters lie mainly in peripheral communities; high-high clusters of affordability/price changes are more spatially dispersed, and availability changes show weaker local clustering.

**Table A7:** Global Moran’s I for representative indicators (KNN weights, 999 permutations).

Indicator	k	n	Moran’s I	Permutation *p*
Δ accessibility, Japanese cuisine	6	3737	0.989	0.001
Δ accessibility, Japanese cuisine	8	3737	0.987	0.001
Δ accessibility, rice noodles and wheat noodles	6	4535	0.774	0.001
Δ accessibility, rice noodles and wheat noodles	8	4535	0.774	0.001
Δ affordability, Jiangsu–Zhejiang cuisine	6	4487	0.507	0.001
Δ affordability, Jiangsu–Zhejiang cuisine	8	4487	0.483	0.001
Δ availability, rice set meals	6	4561	0.082	0.001
Δ availability, rice set meals	8	4561	0.082	0.001
Accessibility level, Japanese cuisine (OFDS-FE)	6	4372	0.994	0.001
Accessibility level, Japanese cuisine (OFDS-FE)	8	4372	0.993	0.001
Affordability level, Jiangsu–Zhejiang cuisine (OFDS-FE)	6	4626	0.682	0.001
Affordability level, Jiangsu–Zhejiang cuisine (OFDS-FE)	8	4626	0.676	0.001

**Table A8:** Cluster-by-category means and medians of the three indicators in the W-FE and the OFDS-FE (medians in parentheses). Full P10–P90 percentile ranges are available from the corresponding author on request.

Dimension	Category	Environment	All	Cluster 0	Cluster 1	Cluster 2	Cluster 3	Cluster 4	Cluster 5	Cluster 6
Accessibility	Jiangsu-Zhejiang cuisine	W-FE	10.7 (8.4)	10.2 (8.2)	10.7 (8.9)	13.7 (10.3)	14.7 (11.7)	18.6 (12.9)	6.9 (4.2)	6.5 (4.4)
Accessibility	Jiangsu-Zhejiang cuisine	OFDS-FE	50.8 (42.4)	43.5 (37.0)	47.1 (40.5)	88.7 (88.4)	106.9 (106.7)	112.0 (114.6)	20.4 (16.8)	14.7 (9.4)
Accessibility	Sichuan-Hunan cuisine	W-FE	10.7 (8.4)	10.0 (8.0)	10.8 (9.1)	13.7 (10.3)	14.7 (11.7)	18.6 (12.9)	7.0 (3.8)	6.7 (4.6)
Accessibility	Sichuan-Hunan cuisine	OFDS-FE	57.1 (62.2)	54.4 (57.1)	60.3 (60.9)	76.3 (78.5)	85.5 (86.4)	83.9 (83.6)	32.0 (25.9)	25.0 (14.9)
Accessibility	Japanese cuisine	W-FE	6.2 (3.2)	4.8 (2.5)	5.2 (3.2)	9.7 (4.9)	11.6 (6.0)	16.9 (9.3)	2.5 (1.5)	1.8 (1.0)
Accessibility	Japanese cuisine	OFDS-FE	17.5 (14.5)	14.1 (10.3)	16.4 (13.5)	30.3 (30.7)	38.4 (38.8)	37.5 (37.8)	4.4 (3.9)	3.8 (2.0)
Accessibility	Rice noodles and wheat noodles	W-FE	10.8 (8.8)	9.9 (7.9)	10.4 (8.6)	13.9 (11.4)	17.5 (15.2)	17.8 (14.7)	9.3 (8.2)	7.1 (5.5)
Accessibility	Rice noodles and wheat noodles	OFDS-FE	155.0 (163.5)	147.8 (150.1)	162.3 (168.1)	206.5 (216.0)	229.8 (230.5)	216.6 (222.3)	101.1 (89.9)	71.9 (51.3)
Accessibility	Rice set meals	W-FE	18.5 (15.1)	17.2 (13.8)	18.0 (15.3)	24.7 (21.0)	30.7 (29.0)	31.1 (29.3)	13.2 (10.3)	11.4 (9.1)
Accessibility	Rice set meals	OFDS-FE	161.3 (174.2)	157.5 (165.5)	172.9 (179.4)	202.0 (210.9)	221.4 (223.2)	208.8 (217.1)	115.3 (96.0)	71.2 (40.0)
Accessibility	Braised and marinated deli foods	W-FE	4.0 (3.3)	3.7 (3.1)	3.6 (3.1)	5.6 (4.9)	6.1 (6.0)	5.7 (5.1)	3.5 (3.0)	3.0 (2.6)
Accessibility	Braised and marinated deli foods	OFDS-FE	60.2 (61.6)	56.2 (54.8)	61.0 (61.0)	87.4 (90.5)	103.3 (101.5)	99.4 (98.3)	34.3 (32.4)	22.6 (12.8)
Availability	Jiangsu-Zhejiang cuisine	W-FE	0.465 (0.198)	0.509 (0.198)	0.537 (0.266)	0.202 (0.104)	0.303 (0.207)	0.227 (0.139)	0.519 (0.269)	0.586 (0.231)
Availability	Jiangsu-Zhejiang cuisine	OFDS-FE	0.234 (0.144)	0.243 (0.138)	0.254 (0.184)	0.163 (0.095)	0.300 (0.194)	0.194 (0.085)	0.227 (0.182)	0.242 (0.114)
Availability	Sichuan-Hunan cuisine	W-FE	0.465 (0.198)	0.501 (0.192)	0.559 (0.288)	0.202 (0.104)	0.303 (0.207)	0.227 (0.139)	0.568 (0.284)	0.511 (0.184)
Availability	Sichuan-Hunan cuisine	OFDS-FE	0.342 (0.186)	0.371 (0.186)	0.366 (0.270)	0.154 (0.074)	0.239 (0.150)	0.154 (0.062)	0.434 (0.320)	0.485 (0.174)
Availability	Japanese cuisine	W-FE	0.160 (0.071)	0.150 (0.061)	0.201 (0.099)	0.123 (0.063)	0.234 (0.115)	0.177 (0.104)	0.171 (0.080)	0.064 (0.030)
Availability	Japanese cuisine	OFDS-FE	0.067 (0.034)	0.065 (0.029)	0.082 (0.051)	0.053 (0.029)	0.105 (0.070)	0.066 (0.027)	0.048 (0.026)	0.031 (0.012)
Availability	Rice noodles and wheat noodles	W-FE	1.315 (0.647)	1.355 (0.650)	1.533 (0.841)	0.747 (0.368)	1.148 (0.609)	0.802 (0.424)	1.541 (0.729)	1.447 (0.868)
Availability	Rice noodles and wheat noodles	OFDS-FE	0.974 (0.529)	1.014 (0.569)	1.075 (0.708)	0.519 (0.226)	0.638 (0.354)	0.442 (0.167)	1.108 (0.623)	1.413 (0.760)
Availability	Rice set meals	W-FE	2.321 (1.067)	2.489 (1.054)	2.778 (1.391)	1.324 (0.697)	1.890 (0.982)	1.401 (0.738)	2.426 (1.415)	2.103 (1.052)
Availability	Rice set meals	OFDS-FE	1.031 (0.537)	1.101 (0.557)	1.202 (0.734)	0.441 (0.220)	0.625 (0.335)	0.415 (0.158)	1.402 (0.918)	1.199 (0.603)
Availability	Braised and marinated deli foods	W-FE	0.426 (0.202)	0.458 (0.199)	0.468 (0.270)	0.262 (0.118)	0.379 (0.214)	0.267 (0.102)	0.550 (0.370)	0.433 (0.196)
Availability	Braised and marinated deli foods	OFDS-FE	0.345 (0.200)	0.384 (0.203)	0.360 (0.284)	0.167 (0.082)	0.286 (0.193)	0.182 (0.070)	0.461 (0.338)	0.412 (0.193)
Affordability	Jiangsu-Zhejiang cuisine	W-FE	2.74 (2.67)	2.80 (2.71)	2.81 (2.70)	2.59 (2.59)	2.58 (2.60)	2.31 (2.26)	2.78 (2.66)	2.76 (2.68)
Affordability	Jiangsu-Zhejiang cuisine	OFDS-FE	3.92 (3.87)	3.94 (3.88)	3.93 (3.89)	3.85 (3.82)	3.82 (3.77)	3.80 (3.77)	4.03 (4.06)	3.96 (3.90)
Affordability	Sichuan-Hunan cuisine	W-FE	2.74 (2.67)	2.81 (2.71)	2.78 (2.70)	2.59 (2.59)	2.58 (2.60)	2.31 (2.26)	2.81 (2.68)	2.76 (2.69)
Affordability	Sichuan-Hunan cuisine	OFDS-FE	4.72 (4.72)	4.78 (4.78)	4.77 (4.77)	4.64 (4.61)	4.66 (4.60)	4.58 (4.54)	4.74 (4.85)	4.50 (4.50)
Affordability	Japanese cuisine	W-FE	1.43 (1.19)	1.55 (1.28)	1.46 (1.26)	1.03 (0.95)	1.11 (1.01)	0.86 (0.82)	1.84 (1.42)	1.89 (1.39)
Affordability	Japanese cuisine	OFDS-FE	3.25 (2.88)	3.34 (3.04)	3.20 (3.03)	2.62 (2.58)	2.54 (2.49)	2.55 (2.51)	4.11 (3.35)	4.77 (4.17)
Affordability	Rice noodles and wheat noodles	W-FE	6.10 (6.07)	6.16 (6.14)	6.12 (6.11)	5.75 (5.74)	5.68 (5.65)	5.51 (5.49)	6.44 (6.47)	6.42 (6.47)
Affordability	Rice noodles and wheat noodles	OFDS-FE	5.60 (5.60)	5.63 (5.64)	5.67 (5.65)	5.48 (5.40)	5.42 (5.36)	5.39 (5.33)	5.68 (5.70)	5.49 (5.57)
Affordability	Rice set meals	W-FE	6.14 (6.01)	6.22 (6.06)	6.12 (5.99)	6.00 (5.95)	5.74 (5.72)	5.82 (5.81)	6.22 (6.12)	6.23 (6.02)
Affordability	Rice set meals	OFDS-FE	5.69 (5.71)	5.74 (5.75)	5.74 (5.73)	5.60 (5.57)	5.57 (5.56)	5.55 (5.51)	5.84 (5.84)	5.49 (5.55)
Affordability	Braised and marinated deli foods	W-FE	4.85 (4.81)	4.91 (4.85)	4.88 (4.87)	4.61 (4.62)	4.42 (4.42)	4.57 (4.50)	5.02 (4.90)	5.06 (4.91)
Affordability	Braised and marinated deli foods	OFDS-FE	3.81 (3.79)	3.84 (3.83)	3.84 (3.85)	3.68 (3.67)	3.65 (3.63)	3.65 (3.63)	3.91 (3.85)	3.81 (3.83)

## Appendix G. Robustness of the Community Classification

Appendix G documents the robustness checks behind the interpretation of the seven community groups as descriptive strata (Section 4.3). Table A9 summarizes internal validity and reproducibility diagnostics from the original pipeline and from an independent re-implementation. Silhouette scores in the embedding space are modest (0.2–0.3), agreement with a k-means (k = 7) baseline is partial (adjusted Rand index approximately 0.34–0.38), and perplexity perturbations shift boundary assignments (ARI 0.21–0.82 across settings), confirming that the seven groups are not compact natural clusters; with fixed seeds and PCA initialization the solution is exactly reproducible.

**Table A9:** Clustering diagnostics: original pipeline versus independent re-implementation.

Diagnostic	Original Pipeline	Independent Replication
Silhouette (embedding space)	0.2–0.3	0.26–0.31 (perp 30–50)
K-means(k = 7) baseline agreement (ARI)	0.38	0.34
Perplexity perturbation, main groups (ARI)	0.35–0.52 (perp 30–50)	0.21–0.55 vs. paper; 0.33–0.82 between runs
Fixed-seed reproducibility	ARI = 1.0 (identical)	deterministic by construction (init = pca)
Cluster count across settings	7 (eps = 4.2, MinPts = 25, Kneedle)	4–7 (Kneedle eps per embedding: 5.1–6.1)
Conclusion-level check (gradient sign & KW significance under alternative 7-way partition)	—	identical signs, all KW *p* < 0.001 (Table A10)

What matters for the substantive conclusions is whether they survive a different partition. Table A10 therefore re-evaluates the core cluster-level results under an alternative seven-way partition generated with different embedding settings. For all representative indicators, the sign of the centre–periphery gradient (Spearman correlation between distance to the city centre and the indicator change) is identical under both partitions, and Kruskal–Wallis tests of cluster differences remain significant (all *p* < 0.01). Cluster boundary assignments are therefore parameter-sensitive, but the gradient direction and cluster-level conclusions reported in Section 5 are not.

**Table A10:** Conclusion-level check under an alternative seven-way partition. Gradient ρ = Spearman correlation between distance to the city centre and the indicator change across strata.

Indicator	Partition	Strata	Gradient ρ	Kruskal–Wallis *p*
Δ accessibility, Japanese cuisine	paper	7	−0.964	<0.001
Δ accessibility, Japanese cuisine	alternative	7	−0.893	<0.001
Δ accessibility, rice noodles and wheat noodles	paper	7	−0.964	<0.001
Δ accessibility, rice noodles and wheat noodles	alternative	7	−0.893	<0.001
Δ affordability, Jiangsu–Zhejiang cuisine	paper	7	−0.214	<0.001
Δ affordability, Jiangsu–Zhejiang cuisine	alternative	7	−0.964	<0.001
Δ availability, rice set meals	paper	7	0.714	<0.001
Δ availability, rice set meals	alternative	7	0.393	0.0052

## Appendix H. Network-Distance Validation of Walkable Food-Environment Measures

Appendix H evaluates how Euclidean walking buffers affect W-FE estimates. On a stratified subsample of 300 communities covering all seven community clusters, walking-network distances were computed from a pedestrian-filtered municipal road network with 57,011 retained road records, 305,932 nodes and 338,529 undirected segments. The network/Euclidean detour ratio has a median of 1.51 (IQR 1.36–1.81), and 56.0% of restaurant-community pairs within the Euclidean walking thresholds exceed those thresholds when measured by network distance; 6.1% were unreachable in the filtered network. Network-based W-FE accessibility is 33.6–42.9% of the Euclidean level by category, but community rankings remain strongly correlated (Spearman rho = 0.825–0.920 by category and 0.895 overall), and cluster-category mean ordering is preserved (rho = 0.972). Euclidean buffers therefore overstate absolute walkable access while preserving the relative community and cluster patterns used in the main analysis.
